# Combined action observation and motor imagery practice for upper limb recovery following stroke: a systematic review and meta-analysis

**DOI:** 10.3389/fneur.2025.1567421

**Published:** 2025-07-23

**Authors:** Dan Lin, Daniel Lloyd Eaves, John Derek Franklin, Jonathan Richard Robinson, Jack Aaron Binks, Jonathan Reyes Emerson

**Affiliations:** ^1^School of Health and Life Sciences, Teesside University, Middlesbrough, United Kingdom; ^2^Biomedical, Nutritional and Sport Sciences, Faculty of Medical Sciences, Newcastle University, Newcastle, United Kingdom; ^3^Population Health Sciences, Faculty of Medical Sciences, Newcastle University, Newcastle, United Kingdom; ^4^Kleijnen Systematic Reviews (KSR) Ltd, York, United Kingdom

**Keywords:** combined action observation and motor imagery, mental practice, stroke survivors, stroke rehabilitation, upper limb recovery, mirror neurons, imitation learning, neuroplasticity

## Abstract

**Introduction:**

Optimal upper limb recovery requires high-dose physiotherapy; however, this essential component of rehabilitation is under-delivered. Mental practice represents an accessible and cost-effective adjunct to conventional therapy. We therefore evaluated the efficacy of an enhanced mental practice treatment (combined action observation and motor imagery, AO + MI) for promoting upper limb recovery post stroke.

**Methods:**

Searching 10 databases, we identified 18 eligible studies (*N* = 336), comprising nine randomized controlled trials (RCTs) and nine non-randomized controlled trials (non-RCTs). RCTs were meta-analyzed using upper limb function outcomes (Fugl-Meyer Assessment for upper extremity, FMA-UE; Action Research Arm Test, ARAT). Non-RCTs (not eligible for meta-analysis) were narratively synthesized using upper limb and neuroimaging outcomes.

**Results:**

Seven RCTs reported FMA-UE scores (*n* = 189), where the standardized mean difference (SMD) for AO + MI treatments was moderate (SMD = 0.58, 95%CI: 0.13–1.04, *p* = 0.02). Two additional RCTs reported ARAT scores. Meta-analyzing the combined FMA-UE and ARAT scores (*n* = 239) revealed SMD = 0.70 (95%CI: 0.32–1.09, *p* = 0.003). No significant correlations existed between the pooled effect size and several moderators (age, time since stroke, intervention duration, control condition, outcome measure and AO + MI arrangement), indicating consistent AO + MI practice effects. Overall, AO + MI significantly improved upper limb function across all nine RCTs, and all nine narratively synthesized studies, including neuroimaging outcomes. Limitations included inconsistent terminology, intervention design, clarity of reporting, and modality.

**Discussion:**

AO + MI practice can promote upper limb recovery following stroke. AO + MI can therefore be used as a bridge between AO therapy (requiring little effort in early recovery), and the more cognitively demanding MI. Researchers must adopt standardized reporting protocols to further establish AO + MI practice efficacy.

**Systematic review registration:**

The review was registered with PROSPERO under the registration number CRD42023418370. The registration is publicly accessible at the following URL: https://www.crd.york.ac.uk/PROSPERO/view/CRD42023418370.

## 1 Introduction

A stroke is a medical emergency resulting from the interruption or reduction of blood flow to the brain, depriving brain tissue of oxygen and nutrients, leading to rapid cell death (1). It is a major cause of long-term disability, with nearly 50% of survivors experiencing persistent impairments in mobility, speech, and cognition (2). Functional and cognitive impairments, including dementia, are also common following a stroke, with their incidence rising over extended follow-up periods (3). These functional deficits significantly reduce the quality of life and create substantial burdens on caregivers and healthcare systems (4). Upper limb impairment is among the most debilitating consequences of a stroke, significantly limiting a survivor’s ability to perform daily activities and achieve functional independence (5). This impairment, which affects the majority of stroke survivors, often results in long-term disability, diminished independence, and a reduced quality of life (6, 7).

The significant impact of upper limb impairment underscores the need for effective rehabilitation strategies to restore motor function and support recovery. High-dose physiotherapy interventions are evidence-based and essential for optimizing recovery outcomes (8, 9). According to the National Institute for Health and Care Excellence (NICE) quality standards, individuals receiving stroke rehabilitation in hospital or community settings should receive a minimum of 45 min of relevant physiotherapy 5 days a week, alongside regular evaluations with professionals and vocational rehabilitation (10). Although the percentage of patients receiving this level of treatment has increased over the past 5 years, a significant number of individuals still do not receive the necessary amount of rehabilitation therapy (10). Furthermore, Veerbeek et al. (11) highlighted routine physiotherapy, which forms the foundation of post-stroke rehabilitation, often falls short of meeting the intensity needed to achieve optimal results. Therefore, exploring accessible, low-cost, and effective adjunct interventions is essential to promoting stroke recovery.

Mental practice is recognized as an intervention for promoting upper limb recovery following stroke (12). It is therefore recommended for use by several international guidelines for achieving best practice in stroke recovery (10, 13–15). For example, “People with stroke who are able and motivated to participate in the mental practice of an activity should be offered training and encouraged to use it to improve arm function, as an adjunct to usual therapy.” (p. 32, 118). This is a low-cost, accessible and non-invasive approach that can complement physiotherapy to facilitate motor recovery (16).

Traditionally, there have been two forms of mental practice used for promoting upper limb recovery post stroke: action observation (AO) and motor imagery (MI) (17, 18). AO is a form of mental practice involving the deliberate and structured observation of another person performing a movement task or skill (19). This can enhance motor learning by activating, in the observer, an internal motor representation of the observed action, and thereby facilitating action imitation (20–22, 119).

Research indicates that AO therapy can significantly improve upper limb movements in ischemic stroke patients by stimulating neural circuits involved in action execution and motor learning (23–25). While those systematic reviews and meta-analyses found beneficial effects for AO therapy on upper limb recovery in stroke survivors, the magnitude of AO effects is often small, and can be below clinical thresholds of detection [see (26)]. Since AO requires little effort on behalf of the user, this technique may be best suited to early stages of recovery.

MI is the mental simulation of a movement without physical execution, involving the visualization of oneself performing a task along with the kinesthetic aspects associated with performing that action (27). This process activates neural pathways similar to those engaged during actual movement, emphasizing the internal cognitive mechanisms that promote neural activation associated with physical performance (28, 29).

A substantial body of research indicates that MI can enhance motor abilities in neurorehabilitation, with neural reorganization following MI practice being similar to the changes observed after physical training (30–32). Studies demonstrate that MI promotes neuroplasticity effects in stroke patients and that acute MI modulates plasticity at both the cortical level and the level of spinal presynaptic inhibition, underscoring the sensitivity of spinal circuitry to MI (33, 34).

MI is easily integrated into conventional physiotherapy for stroke rehabilitation (35). Recent reviews have highlighted the effectiveness of MI in reducing upper limb activity limitations following stroke [e.g., (36)]; particularly during the initial 3 months post-stroke, and in individuals experiencing the most severe upper limb dysfunction (37). Stroke patients can however encounter difficulties generating MI from verbal instructions in clinical settings, due to cognitive impairments that affect their ability to follow such cues (38). Moreover, MI ability can either be significantly impaired or absent following damage to the inferior parietal lobe (39).

Previous research has primarily used either AO or MI in isolation or in comparison. Over the past decade, however, there has been a growing interest in the potential advantages of combining these two approaches into a single intervention, known as combined action observation and motor imagery [AO + MI, (18, 40)]. This method involves the user observing a movement (e.g., via a video or live demonstration) while simultaneously self-generating an imagined kinesthetic representation of the same action and synchronizing this imagery with the observed action (18, 40). Multimodal brain imaging studies provide robust and consistent evidence in healthy adults that AO + MI practice significantly increases neurophysiological activation in cortico-motor regions of the brain, exceeding the involvement that occurs via either AO or MI separately [e.g., (41–44, 120); see (45, 46)].

From a practical viewpoint, the visual display directly specifies the kinematic features of the to-be-imagined action (such as hand velocity and trajectory). The AO component of AO + MI can therefore help to reduce the cognitive load associated with self-generating the MI content (47). This will be particularly relevant to stroke survivors with specific cognitive dysfunction that impairs their ability to either generate or maintain MI (38).

While there is robust evidence supporting the efficacy of AO + MI practice on motor outcomes in healthy adults (46, 48), the early clinical studies in stroke rehabilitation also show promising results (49–51). These studies show AO + MI practice can improve upper limb motor function in stroke patients, despite relatively small sample sizes. For example, AO + MI practice enhanced pinch-grip strength and dexterity in the affected limb, while yielding more pronounced changes in the activation of motor-cortical brain regions compared to control conditions (49, 51).

Across several clinical studies, there has been a proliferation of terminology used to describe AO + MI interventions across modalities, including virtual reality [VR, (52, 53)] and brain-computer interfaces [BCIs, (54)]. Sub-categories have also emerged [e.g., (51)], whereby AO + MI practice is delivered either synchronously, where AO and MI are performed at the same time, or asynchronously, where AO and MI are presented separately (i.e., alternating AO followed by MI). Currently, however, the optimal delivery mode remains unclear. A systematic review and meta-analysis of the available studies is now warranted to determine the true effects of AO + MI practice in stroke survivors.

Despite the optimistic findings reported, the overall effectiveness of AO + MI in enhancing motor function during stroke rehabilitation remains uncertain. Recent work has questioned the efficacy of AO + MI for improving lower-limb motor function, highlighting the need for further investigation (55). It is now pertinent to identify key factors influencing treatment outcomes, including time since stroke, age, intervention duration, technology used, and patients’ prior experiences with similar rehabilitation techniques. Understanding these variables is critical for optimizing AO + MI interventions and customizing them to meet individual patient needs, ultimately to enhance recovery.

We conducted a systematic review and meta-analysis on eligible randomized control trial (RCT) studies that investigated AO + MI practice effects in stroke survivors and reported standardized outcome measures for motor function. The aim was to evaluate the efficacy of AO + MI practice, specifically for upper limb recovery in stroke patients. We also explored several moderators that may influence treatment outcomes. We further conducted a narrative synthesis of non-RCT design AO + MI practice studies that reported behavioral and neurophysiological measures (of brain function) with regards to motor recovery. By synthesizing the existing evidence, this study sought to clarify the role of AO + MI in stroke rehabilitation and contribute to the optimization of rehabilitation protocols tailored to individual patient needs.

## 2 Methodology

This review followed the methodological guidance and reporting standards for systematic reviews and meta-analyses as outlined in the PRISMA guidelines and the Cochrane Handbook (56, 57). Additionally, the review was registered with PROSPERO under the registration number CRD42023418370.

### 2.1 Eligibility criteria

The decision to include a study was based on five criteria aligned with the PICOS framework: P (Population), I (Intervention), C (Comparator), O (Outcomes), and S (Study Design).

Population: Participants were required to be adults aged 18 years and older with a confirmed diagnosis of stroke, either through clinical criteria or diagnostic imaging.

Intervention: Studies had to include an AO + MI intervention (delivered either synchronously or asynchronously), that targeted upper limb recovery, with no restrictions on the type of MI instructed (visual or kinesthetic), or on the strategies employed to instruct mental practice, such as videos, images, audio recordings, virtual reality (VR) or brain-computer interfaces (BCIs). While our aim was to quantify the isolated effect of AO + MI practice, studies were included if the AO + MI component could be reasonably separated from other therapeutic elements through the design of the control condition. For example, interventions combining AO + MI with BCIs were included if the control group allowed the specific contribution of AO + MI to be inferred. Conversely, studies were excluded if AO + MI was part of a complex intervention where its effect could not be separated.

Comparison: The study had to include a control or comparison group that received conventional therapy, usual care, alternative intervention, or no therapy.

Outcome: The study had to evaluate the effects of AO + MI practice on clinical assessments of upper limb function, using commonly employed measures. The primary outcomes selected for inclusion in the meta-analysis were the Fugl-Meyer Assessment Upper Extremity (FMA-UE) and the Action Research Arm Test (ARAT). The secondary outcomes included the Wolf Motor Function Test (WMFT), Motor Activity Log (MAL), Pinch Strength Test (PST), Box and Block Test (BBT), grip strength, the Nine-Hole Peg Test (9-HPT) and electromyography (EMG, e.g., spectral power). Additionally, studies could assess neurophysiological outcomes, also categorized as secondary, using brain imaging tools such as electroencephalography (EEG, e.g., event-related desynchronization, ERD), transcranial magnetic stimulation (TMS, e.g., motor-evoked potentials, MEP), functional near-infrared spectroscopy (fNIRS, e.g., oxyhemoglobin and deoxyhemoglobin levels), functional magnetic resonance imaging (fMRI, e.g., blood oxygenation level distribution, BOLD).

Study design: The included studies encompassed any experimental design, including randomized controlled trials (RCTs), non-randomized studies (non-RCTs) with a control or comparison group, and single-group pre-post or within-subject designs. These studies compared the effectiveness of AO + MI practice in stroke rehabilitation to other approaches, such as independent AO or MI, or conventional rehabilitation.

### 2.2 Search strategy and study selection

A structured search strategy was developed in collaboration with an academic librarian at Teesside University to ensure comprehensive coverage of the relevant literature (see Supplementary materials). This strategy incorporated a combination of keywords and Medical Subject Heading (MeSH) terms to improve the precision and broaden the scope of the search. The search was conducted across multiple databases, including MEDLINE, AMED, Web of Science, Embase, CINAHL, PsycINFO, PEDro, PubMed, Scopus, and the Cochrane Library. The initial search was conducted in June 2023, with the final search completed in March 2024. Consistent search terms were established and tailored for each database according to the specific requirements of the search strings. The search was restricted to studies published in English, with no limitations on the years of publication. To further ensure thoroughness, the database search was supplemented with hand searching and citation tracking. In addition to database searches, we explored gray literature sources to reduce potential publication bias. These included clinical trial registries (e.g., ClinicalTrials.gov, National Research Register, Mata-Register of Controlled Trials, Clinical Trials), conference abstracts, and dissertations (e.g., EThOS). However, no additional eligible studies were identified through these sources. Several trial registry entries lacked posted results or did not meet the inclusion criteria.

### 2.3 Screening process

This study conducted a two-stage screening process, comprising title and abstract screening followed by full-text screening. The titles and abstracts of the articles were independently screened by two reviewers (DL and JE) to assess their eligibility. During this process, studies were categorized as “include,” “maybe,” or “exclude.” All included and maybe papers were involved in the second selection process which was based on reading the full texts. The same reviewers independently evaluated the full-text articles. Any disagreements that arose during this process were resolved through discussion. If consensus could not be reached, an independent reviewer (DE) was consulted to provide a final judgement. The screening processes were conducted using the web tool Rayyan (58), which facilitates search exploration, saves time, and simplifies the sharing and comparison of inclusion and exclusion decisions.

### 2.4 Data extraction

A data extraction form was developed and piloted based on five included studies. The extracted data encompassed various domains, including study characteristics, participant demographics, details of the intervention, and disease-specific factors such as stroke type and time since stroke. The extraction of intervention details adhered to the guidelines outlined in the Template for Intervention Description and Replication (TiDIER) checklist (59). Data on upper limb function, including means, standard deviations (SD), and sample sizes, were extracted from the included RCTs for use in the meta-analyses. For each included non-RCTs, data on upper limb function and neuroimaging were extracted as reported, including effect sizes and sample sizes. Given the substantial variability in non-RCTs, information on study characteristics (e.g., study design, timing of assessment, and intervention protocol) was also extracted to support a more contextual narrative synthesis. Data extraction was conducted independently by two reviewers (DL and JE), with any inconsistencies resolved through discussion or consultation with a third reviewer (DE).

### 2.5 Effect size preparation

In the meta-analysis of upper limb function outcomes, effect sizes were assessed using the standardized mean difference (Cohen’s *d*). Cohen’s *d* was calculated based on the mean, SD, and sample size values, specifically by dividing the mean difference by the pooled SD (60, 61). Cohen’s *d* is employed to standardize results across a common scale when studies assess the same outcome but utilize different measurement instruments; for instance, both the FMA-UE and ARAT assess upper limb function in stroke survivors but employ distinct measurement scales, making standardization necessary for comparison. Cohen’s *d* effect size values were converted to Hedge’s *g* using a small sample size correction formula to enhance accuracy in the sensitivity analyses (60, 62). Cohen’s *d* and Hedge’s *g* were calculated using the following Equations 1, 2:


(1) $$d=\frac{M_{1}-M_{2}}{SD_{\text{pooled}}}\;$$

(2) $$g=d\times (1-\frac{3}{4N-9})\;$$

In these two equations, *d* represents Cohen’s *d*, M_1_ denotes the mean of the experimental group, M_2_ denotes the mean of the control group, *SD_pooled_* represents the pooled SD of the two groups, *g* indicates Hedge’s *g*, and *N* refers to the total number of participants across both groups (60, 62).

The effect size calculation process differed for five studies due to variations in the types of data reported. In the study by Sun et al. (51), data were reported across multiple time points. For this meta-analysis, the data collected at the fourth week were selected for calculating the mean change score, as this time point represents the longest follow-up in the study, which served as the post-treatment assessment. This choice follows Cochrane Review recommendations, which suggest selecting the longest time point when multiple outcomes are measured within a timeframe (56). Additionally, this approach aligns with the guideline to choose the most relevant outcome from available options (63). Since other included studies reported post-treatment data, selecting the fourth-week data point ensured consistency and comparability across studies. Additionally, the mean difference (MD) and SD between pre and post within the control and experimental groups were not reported. However, Cohen’s *d* for each time point was provided. Consequently, the MD, standard error (SE) of the MD, pooled SD of Cohen’s *d*, and the SE of Cohen’s *d* between the pre-treatment and week 4 time points were calculated using the following Equations 3–7:


(3) $$\mathrm{MD}=(MD_{E,w4}-MD_{E,\mathrm{pre}})-(MD_{C,w4}-MD_{C,\mathrm{pre}})\;$$

(4) $$d_{W4-\mathrm{pre}}=d_{W4}-d_{\mathrm{pre}}\;$$

(5) $$SD_{W4-\mathrm{pre}}=\frac{\mathrm{MD}}{d_{W4-\mathrm{pre}}}\;$$

(6) $${\mathrm{SE}}_{d}=\sqrt{\frac{\left(n_{1}+n_{2}\right)}{n_{1}n_{2}}+\frac{d^{2}}{2(n_{1}+n_{2})}}\;$$

(7) $$SE_{d,W4-\mathrm{pre}}=\sqrt{S{E^{2}}_{d,\mathrm{pre}}+S{E^{2}}_{d,W4}}\;\;$$

In these formulas, MD indicated the change mean of two groups, MD*_E, W4_* and MD*_E, pre_* denote the experimental group means at week 4 and baseline, respectively, while MD*_C, W4_* and MD*_C,pre_* indicate the control group means at week 4 and baseline, d*_W4-pre_* represents Cohen’s *d* for the mean change between week 4 and baseline, d*_W4_* for the week 4 mean difference, d*_pre_* for the baseline mean difference, SD*_W4-pre_* represents the SD of changes, SE*_d, W4-pre_* represents the SE of changes, and n_1_ and n_2_ are the sample sizes of the experimental and control groups, respectively.

Similar to the study by Sun et al. (51), the study by Timmermans et al. (64) reported data across multiple time points, including baseline, post-training at 6 weeks, and two follow-up assessments at 6- and 12-months post-intervention. For consistency and standardization, the data from the 6-week post-treatment time point were selected for inclusion in this meta-analysis. However, the study reported median and interquartile range data instead of MD and SD. Therefore, the median and interquartile range values were converted to estimates of the sample mean and SD using a three-step process. First, a skewness test was performed using sample size, median, and interquartile range to assess normality (65). Results indicated that the data were not significantly skewed, suggesting that the distribution closely resembles a normal distribution. This allowed us to assume that the mean and median were approximately equal, indicating a balanced distribution around the centre. Next, the sample mean was estimated using the approach proposed by Luo et al. (66), where the sample SD was estimated following the method outlined by Wan et al. (67).

The correlation coefficient *r* value was reported in the study by Green et al. (68) and was used to impute the change-from-baseline SD using the following Equation 8:


(8) $$SD_{\text{change}}=\sqrt{S{D^{2}}_{\text{basedline}}+S{D^{2}}_{\text{final}}-(2\times r\times SD_{\text{baseline}}\times SD_{\text{final}})}\;$$

In this equation, SD*_change_* represents the change-from-baseline SD, SD*_baseline_* denotes the baseline SD, SD*_final_* denotes the final SD, *r* represents the change-from-baseline correlation coefficient (56).

In the study by Liu et al. (69), only the *F*-value for FMA-UE data was reported. The *F*-value was converted to Cohen’s *d* following the guidelines of Lipsey and Wilson (70) and calculated using the Campbell Collaboration effect size calculator (71). Similarly, in the study by Thara et al. (72), the *t*-values for the pre- and post-intervention differences between groups were reported. These *t*-values were converted to Cohen’s *d* and Hedge’s *g* following Lipsey and Wilson (70) and calculated using the same effect size calculator.

For nine non-RCT studies, methodological and outcome differences were too diverse for data pooling and statistical comparison. Such an analysis would invalidate typical methods of standardization (73, 121). A narrative synthesis was therefore the most appropriate method of analysis for those non-RCT design studies.

### 2.6 Quality assessment

The methodological risk of bias was evaluated and reported following the guidelines outlined in the Cochrane Handbook for Systematic Reviews of Interventions (56). Two reviewers (DL and JE) independently assessed the quality of all included studies using the version 2 of the Cochrane risk-of-bias tool (RoB2) for RCTs (74) and the risk of bias in non-randomized studies–of Interventions (ROBINS-I) tool for non-randomized studies (75). The RoB2 tool is a revised instrument specifically designed for evaluating the risk of bias in RCTs, assesses five key domains: the randomization process, deviations from intended interventions, missing outcome data, outcome measurement, and selection of the reported result, providing a structured approach to determine the potential impact of systematic errors on study outcomes.

The ROBINS-I tool is used to assess the risk of bias in non-randomized studies of interventions, evaluating bias across multiple domains such as confounding, participant selection, intervention classification, deviations from intended interventions, missing data, outcome measurement, and reporting, allowing for a nuanced assessment of studies where randomization is not feasible. Both the RoB2 and the ROBINS-I tools are recommended as comprehensive and reliable instruments for assessing the risk of bias in clinical studies, providing structured frameworks for evaluating potential biases that may impact research validity (75, 76). In conjunction with the RoB2 tool, ROBINS-I ensured a comprehensive and standardized evaluation of the included studies.

The quality of evidence for the outcomes included in the meta-analysis was independently evaluated by two reviewers (DL and JE) using the Grading of Recommendations, Assessment, Development, and Evaluations (GRADE) approach (77). Each outcome was assessed across the following domains: risk of bias, inconsistency, imprecision, indirectness, and publication bias (78).

### 2.7 Data analysis

Upper limb function outcomes (FMA-UE and ARAT) from randomized controlled trials were analyzed using a random-effects model. In addition to the primary meta-analysis based solely on FMA-UE scores, a secondary meta-analysis was conducted that included both FMA-UE and ARAT data to explore overall effects across impairment and activity domains. Heterogeneity was estimated with the restricted maximum-likelihood method (79), and tests with confidence intervals were calculated using the Knapp and Hartung method (80). Due to the inability to statistically pool the data and the heterogeneity in study designs and outcomes, non-RCTs and neuroimaging outcomes were synthesized narratively. Statistical analyses were conducted in RStudio using the ‘metafor’ package (v4.2.0). For upper limb function outcomes reported in the nine RCT studies, pooled treatment effects, along with 95% confidence intervals (CIs) and 95% prediction intervals (PIs), were calculated. The degree of heterogeneity was evaluated through visual inspection of forest plots and by calculating the Chi^2^ test, the Tau^2^ statistic and the *I*^2^ (I-squared) statistic.

To address concerns regarding between-study heterogeneity, outlier diagnostics were performed using the ‘FIND.OUTLIERS’ function, and influence analyses were conducted using the ‘INFLUENCEANALYSIS’ function from the ‘dmetar’ package in R (81). This process involved visual inspection of the “Baujat” and “influence” plots for effect sizes, as well as “leave-one-out” plots for both effect size and I^2^ values.

Potential outliers and influential effect sizes were identified across the meta-analysis. The removal of these outliers and influential cases had a minimal impact on the pooled effect and heterogeneity estimates for the meta-analyses. One effect size (82) was excluded from the meta-analysis as it was deemed an influential outlier, resulting in a substantial change to the pooled effect and heterogeneity when removed from the analysis (see Supplementary materials). All other effect sizes were retained in the meta-analyses to maintain the integrity and richness of the data.

Meta-regression analyses were conducted to assess whether moderators influenced the effect of AO + MI practice on upper limb function compared to the aggregate data from control conditions in the meta-analyses. Moderator variables were selected based on theoretical and clinical relevance to stroke recovery and AO + MI interventions. Time since stroke and participant age were included as biological and recovery-related factors that can influence neuroplasticity and responsiveness to therapy (83–85). Intervention duration was assessed due to its known role in dose–response relationships in rehabilitation (86). AO + MI arrangement (synchronous vs. asynchronous) was included to explore potential differences in modalities of action simulation (48). BCI inclusion was used to evaluate the influence of AO + MI interventions supported by this technology vs. those that are not. Control condition type and outcome measure (FMA-UE vs. ARAT) were included because they may confound or moderate observed effects across studies, based on the ICF domain differences and intervention contrast.

A sensitivity analysis was conducted by re-evaluating the meta-analyses using previously calculated Hedge’s *g* values to address concerns regarding sample size bias. A funnel plot was used to evaluate small study bias (publication bias) for outcomes reported in 10 or more studies included in the meta-analysis (87). Additionally, Egger’s statistical test for funnel plot asymmetry was performed to further assess publication bias.

## 3 Results

**Figure 1:**
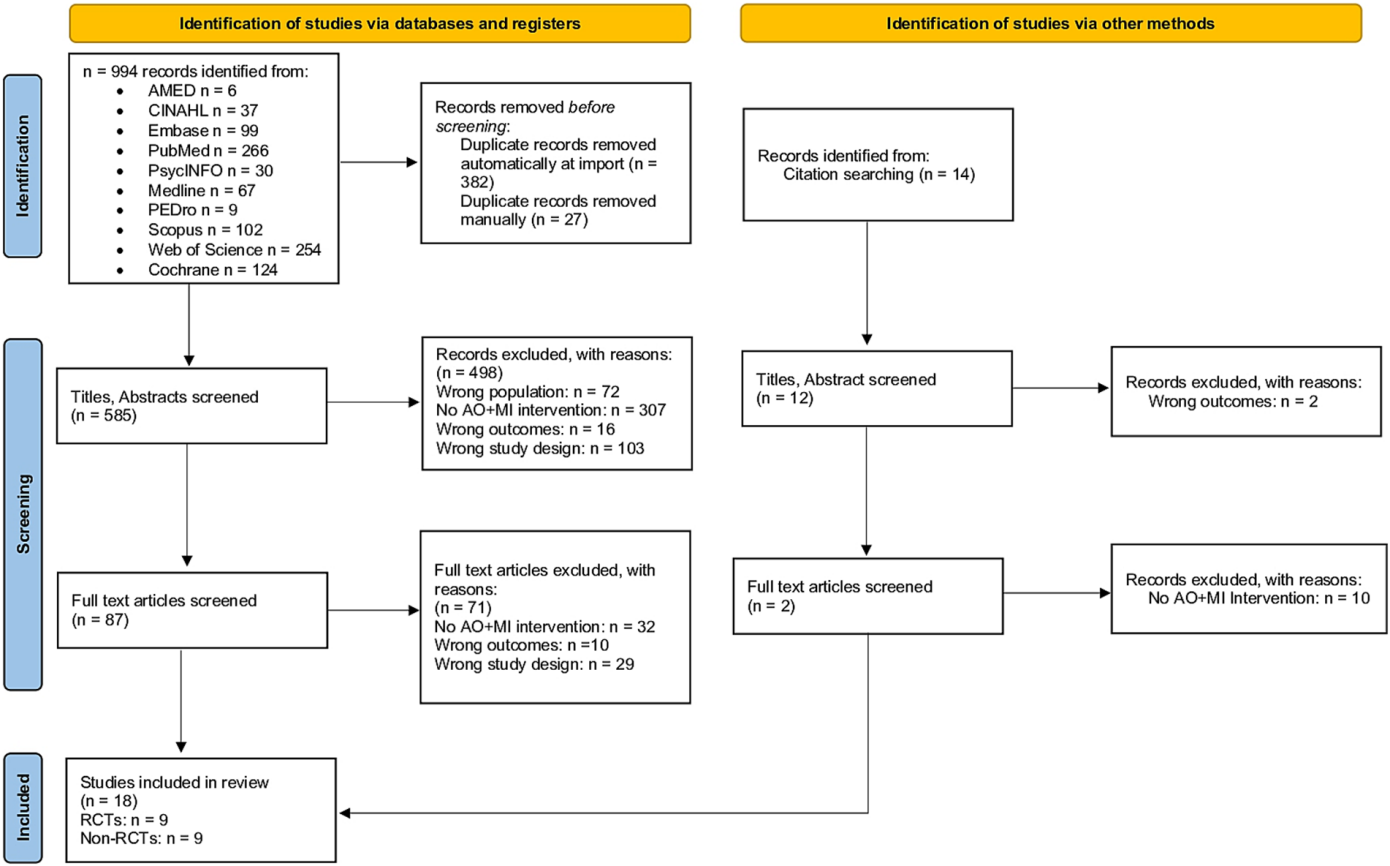
PRISMA flow diagram of study selection. The diagram illustrates the literature search and selection process, showing sources identified, records screened and excluded, and studies included in the final review.

The initial searches yielded 1,008 results, of which 409 were duplicates and subsequently removed. Following the screening of titles and abstracts, 585 unique articles were identified. Among these, 87 full-text articles underwent eligibility assessment, leading to the inclusion of 18 studies in the review (see Figure 1). To obtain additional information regarding the AO + MI intervention, two authors were contacted (82, 88). Both authors responded, providing relevant details. As a result, the final review comprised 18 studies with available data on the AO + MI intervention. Of these studies, nine were RCTs, which were included in the meta-analysis for upper limb function outcomes (49, 51, 54, 68, 69, 72, 88, 89). The remaining nine studies, which were non-RCTs, were analyzed narratively for upper limb function and/or neurophysiological outcomes (52, 53, 90–96).

### 3.1 Study characteristics

This review incorporated a total of 18 studies, comprising nine RCTs and nine non-RCTs, with an aggregate of *N* = 354 participants.

#### 3.1.1 Clinical measure–upper limb function outcomes

Fifteen of the 18 studies (*n* = 326) reported clinical measures for upper limb function (49, 51–54, 64, 68, 69, 72, 88–91, 94, 96). Of these studies, only nine RCTs (*n* = 248) were eligible for meta-analysis. Seven of the nine RCTs (*n* = 189) were analyzed in the first meta-analysis, which included seven effect sizes from seven studies focusing exclusively on FMA-UE outcomes (49, 51, 54, 64, 68, 88, 89). Participants in this analysis had a mean age of 63.8 ± 10.1 years, with 172 inpatients and 17 outpatients. The cohort included *n* = 45 hemorrhagic, *n* = 102 ischemic, and *n* = 62 unspecified strokes, with *n* = 107 in early subacute, *n* = 65 in late subacute, and *n* = 17 in the chronic phase. The AO + MI interventions (*n* = 94) included four studies with synchronous AO + MI (49, 51, 64, 89), two with asynchronous AO + MI (68, 88), and one with synchronous AO + MI-based BCI training (54). All studies focused on upper limb functional training, with session durations 20–60 min, 3–7 sessions per week, and total durations ranging from 3 to 10 weeks (see Table 1).

**Table 1:** Randomized controlled trials.

Study characteristics					
Study	Setting	Participants	Type of intervention	Outcome measure	Summary of results
(51)*	Inpatient	*n* = 10 Age (y): 59.8 ± 4.94 Sex: 6M, 4F Diagnosis: 7 H, 3 I Time since stroke: <2 mos	EG: Synchronous AO + MI CG: Asynchronous AO + MI	FMA-UE PST EEG	Compared to the CG, the EG showed significantly greater improvements in FMA-UE, PST, and ERD (*p* < 0.05).
(49)*	Inpatient	*n* = 45 Age (y): 63.1 ± 8.6 Sex: 24M, 21F Diagnosis: 20H, 25 I Time since stroke: 2–8 mos	EG: Synchronous AO + MI CG: AO	FMA-UE WMFT MAL TMS	The EG showed significant improvements in MEP amplitude, FMA UE scores, and MAL after the intervention compared to pre-intervention and the control group.
(89)	Inpatient	*n* = 20 Age (y): 60.7 ± 12.9 Sex: 10M, 10F Diagnosis: 12 H, 8 I Time since stroke: < 6 mos (70.7 ± 47.3 days)	EG: Synchronous AO (video) + MI CG: Conventional therapy	FMA-UE MFT FIM	Significant differences were found from within both EG and CG for FMA-UE and FIM scores. No significant difference found between EG and CG for FMA-UE (*p* = 0.912) and FIM scores (*p* = 0.481).
(54)*	Outpatient	*n* = 17 Age (y): 61.4 ± 6.5 Sex: 14M, 3F Diagnosis: 6 H, 11 I Time since stroke: > 6 mos (55 ± 53.8 mos)	EG: Synchronous AO + MI-based BCI training via FES CG: MI-based BCI training via FES	FMA-UE EEG	Significant improvements in FMA-UE were observed in both groups (*p* = 0.012, 0.018). Significantly higher improvement in EG for FMA-UE (*p* = 0.022) and ERD (*p* = 0.034, 0.021, 0.038) compared to CG.
(64)	Inpatient	*n* = 32 Age (y): 59.3 ± 7.6 Sex: 26M, 16F Diagnosis: NR Time since stroke: 2–6 weeks	EG: Synchronous AO + MI CG: Usual therapy	FMA-UE WMFT FAT ACC	Improvements were found from within both EG and CG for FMA-UE and WMFT. A significant improvement on FAT from EG. No significant difference in training effects found between EG and CG.
(88)	Inpatient	*n* = 46 Age (y): 71.7 ± 7.3 Sex: 22 M, 24F Diagnosis: 46 I Time since stroke: < 3 mos	EG: Asynchronous AO (pictures, video) + MI CG: Conventional functional training	FMA-UE CTT 7-point Likert scale	Significant better improvement in EG for the performance on the trained tasks. No significant difference in FMA-UE and CTT between 2 groups.
(68)	Inpatient	*n* = 18 Age (y): 61 ± 11 Sex: 7M, 16 11 Diagnosis: 0 H, 18 I Time since stroke: < 1 mos	EG: 1. Asynchronous AO (video) + MI 2. Audio + MI 3. RTP CG: Traditional stroke rehabilitation	FMA-UE WMFT	A significant change in FMA-UE scores and WMFT time scores in the audio MP and traditional therapy groups, but no significant change in the video MP and RTP groups. The medium effect size (r) in all groups indicates that all four groups showed improvements in FM scores and reductions in WMFT time. Among all groups, no statistically significant change was found between pretest and posttest scores for the WMFT functional ability score.
(72)	Inpatient	*n* = 30 Age (y): 52.3 ± 5.9 Sex: 21M, 9F Diagnosis: 30 I Time since stroke: <6 mos	EG: Asynchronous AO (video) + MI CG: Mirror therapy	ARAT	No statistically significant difference in grasp and gross movement between EG and CG. A statistically significant difference in grip, pinch and total score between EG and CG.
(69)*	Inpatient	*n* = 20 Age (y): 51 ± 11.39 Sex: 11M, 9F Diagnosis: NR Time since stroke: < 3 mos (1.87 ± 0.72 mos)	EG: Asynchronous AO (video) + MI CG: AO (video)	ARAT fMRI	Significant better improvement in EG for ARAT (*p* = 0.04), and the activated voxels number in the contralateral SMC.

Intervention characteristics						
Study	Group	Type of intervention	Intervention tasks	Session duration (mins)	Total number of sessions	Intervention time and frequency
(51)*	EG	Synchronous AO + MI with conventional rehabilitation	Inserting and removing pegs	22.5	28	7 days/week 4 consecutive weeks
	CG	Asynchronous AO + MI with conventional rehabilitation	Inserting and removing pegs	22.5	28	7 days / week 4 consecutive weeks
(49)*	EG	Synchronous AO + MI with occupational/physical therapy	10 ADLs (e.g., using chopsticks, pen and hand washing). Participants selected and completed five meaningful activities.	25	40	5 days / week Totally 8 weeks
	CG	AO with occupational/physical therapy	10 ADLs (e.g., using chopsticks, pen and hand washing). Participants selected and completed five meaningful activities.	25	40	5 days/week Totally 8 weeks
(89)	EG	Synchronous AO + MI (video) with conventional rehabilitation	6 tasks derived from the manual function test and the Stroke Upper limb Capacity Scale (e.g., peg-board, holding a cup).	20	20 (MI + AO) with 30 conventional rehabilitations	5 days/week Over 4 weeks
	CG	Conventional rehabilitation	Functional training (e.g., gait training, muscle strengthening)	20	30 conventional rehabilitation	5 days/week Over 4 weeks
(54)*	EG	Synchronous AO + MI-based BCI training via FES	Wrist and hand extensions	44	12	3 days/week 4 consecutive weeks
	CG	MI-based BCI training via FES	Wrist and hand extensions	44	12	3 days/week 4 consecutive weeks
(64)	EG	Synchronous AO + MI (video) with usual therapy	Arm training (e.g., holding paper, grasp of a glass).	30	42	7 days/week Over 6 weeks
	CG	Usual therapy	Arm training (e.g., holding paper, grasp of a glass).	30	42	7 days/week Over 6 weeks
(88)	EG	Asynchronous AO + MI (picture, video) with physiotherapy	Daily living tasks (e.g., upper limb coordination)	60	15	5 days/week Over 3 weeks
	CG	Conventional functional training Physiotherapy	Daily living tasks (e.g., upper limb coordination)	60	15	5 days/week Over 3 weeks
(68)	EG_1_	Asynchronous AO (video) + MI	(a) Wiping a table, (b) Picking up a cup, (c) Brushing hair, (d) Turning the page of a book.	NR	NR	20 MI repetitions and 10 physical practice repetitions of the task/session. 5 day/week (included MP and physical practice 3x/week, and MP only 2 days/week)
	EG_2_	Audio + MI	(a) Wiping a table, (b) Picking up a cup, (c) brushing hair, (d) turning the page of a book.	NR	NR	20 MI repetitions and 10 physical practice repetitions of the task / session. 5 day/week (included MP and physical practice 3x/week, and MP only 2 days/week)
	EG_3_	RTP	(a) Wiping a table, (b) Picking up a cup, (c) Brushing hair, (d) Turning the page of a book.	NR	NR	5 day/week NR: Total intervention duration (posttest within 3 days before discharge)
	CG	Traditional stroke rehabilitation	Range of motion, weight-bearing, massage, modalities, and task-oriented training.	NR	NR	5 day/week NR: Total intervention duration (posttest within 3 days before discharge)
(72)	EG	Asynchronous AO (video) + MI with physical practice	Upper limb ADLs (e.g., picking up cup or phone, writing)	60	30	3 days/week for 10 weeks
	CG	Mirror therapy	Upper limb ADLs (e.g., picking up cup or phone, writing)	60	30	3 days/week for 10 weeks
(69)*	EG	Asynchronous AO (video) + MI with physical practice	flexion/extension of the thumb, abduction/adduction of all digits, making a fist/spreading the hand, moving extended fingers backwards and forwards, and moving the hand between the ulnar and radial deviation	45	20	5 days/week 4 weeks
	CG	AO with physical practice	flexion/extension of the thumb, abduction/adduction of all digits, making a fist/spreading the hand, moving extended fingers backwards and forwards, and moving the hand between the ulnar and radial deviation	45	20	5 days/week 4 weeks

Values are mean ± SD; An asterisk indicates the study includes neuroimaging data. EG indicates experiment group; CG, control group; NR, Not reported; F, female; M, male; mos, months; y, year; I, ischemia; H, hemorrhage; mins, minutes; AO, action observation; MI, motor imagery; AO+MI, Combined action observation and motor imagery; FMA-UE, Fugl-Meyer Assessment Upper Extremity; ARAT, action research arm test; PST, Pinch strength test; WMFT, Wolf Motor Function Test; MAL, Motor Activity Log; MFT, manual function test; FIM, Functional independent measure; BCI, Brain–computer interface; FES, functional electrical stimulation; FAT, Frenchay arm test; ACC, accelerometry; CTT, the Color Trails Test; fMRI, Functional magnetic resonance imaging; ADLs, Activities of daily living; EEG, electroencephalogram; MEP, Motor Evoked Potentials; TMS, Transcranial magnetic stimulation; ERD, event-related desynchronization; RTP, Repetitive task practice.

The second meta-analysis included all nine of the eligible RCT studies. This combined seven effect sizes from FMA-UE outcomes in the seven studies (49, 51, 54, 64, 68, 88, 89), plus two effect sizes from ARAT outcomes in two additional studies (69, 72). Please see Discussion (section 4.3) for commentary on the distinctions and associations between these two assessment tools, with regards to pooling these data for meta-analysis. All studies in the meta-analyses were published between 2004 and 2023. The pooled sample in the second meta-analysis had a mean age of 61.3 ± 9.8 years. Eight studies focused on inpatients (*n* = 222) and one on outpatients (*n* = 17). The cohort included *n* = 45 with hemorrhagic stroke, *n* = 132 with ischemic stroke, and *n* = 62 with unspecified strokes. Time since stroke varied: four studies (*n* = 127) were early subacute (<3 months), three studies (*n* = 95) were late subacute (3–6 months), and one study (*n* = 17) were chronic (>6 months).

The interventions varied in terminology and AO + MI arrangement (*n* = 119): four studies used synchronous AO + MI (49, 51, 64, 89) and four used asynchronous AO + MI (68, 69, 72, 88). One study focused on synchronous AO + MI-based brain-computer interface (BCI) training (54).

All interventions involved AO + MI practice of upper limb function movements and activities, including inserting and removing pegs (51); ten activities of daily living (ADLs, e.g., using chopsticks, pen and hand washing) (49); six tasks derived from the manual function test and the Stroke Upper limb Capacity Scale (e.g., peg-board, holding a cup) (89); wrist and hand extensions (54); arm training [e.g., holding paper, grasp of a glass, (64)]; daily living tasks [e.g., upper limb coordination, (88)]; four tasks, e.g., wiping a table, picking up a cup, brushing hair and turning the page of a book (68); upper limb ADLs [e.g., picking up cup or phone, writing, (72)]; flexion/extension of the thumb, abduction/adduction of all digits, making a fist/spreading the hand, moving extended fingers backwards and forwards, and moving the hand between the ulnar and radial deviation (69). Session durations ranged from 20 to 60 min, with 3–7 sessions per week, and total intervention durations ranged from 3 to 10 weeks (see Table 1).

Regarding the 15 studies that reported upper limb function outcomes, six non-RCTs (*n* = 78) that had not been included in the meta-analyses were subjected to a narrative synthesis (52, 53, 90, 91, 94, 96). Participants had a mean age of 65.2 ± 12.7 years, comprising *n* = 68 inpatients and *n* = 10 outpatients. The cohort included *n* = 22 individuals with hemorrhagic stroke, *n* = 50 with ischemic stroke, and *n* = 6 with unspecified strokes. Time since stroke varied among participants, with 51 classified as early subacute, 2 as late subacute, and 25 as chronic. The interventions were AO, MI, synchronous AO + MI, and asynchronous AO + MI, all involving mental practice of an upper limb functional task. Session durations ranged from 1.7–30 min, with a total of 1–58 sessions, and total intervention durations varied from 1 day–5 weeks (see Table 2).

**Table 2:** Non-randomized controlled trials.

Study characteristics						
Study	Study design	Setting	Participants	Type of intervention	Outcome measure	Results
(53)*	Single-group, within-subjects design	Inpatient	*n* = 8 Age (y): 64.8 ± 10.3 Sex: 7 M, 1 F Diagnosis: 8 I, 0 H Time since stroke: 11.12 ± 23.85 mos	Int1: MI Int2: AO Int3: Synchronous AO + MI	EMG EEG	MI + AO produces greater alpha band ERD and a more noticeable beta band ERD compared to MI and AO alone. EMG analysis shows that muscle strength from MI + AO is the highest.
(52)	Single-group, within-subjects design	Inpatient	*n* = 4 Age (y): 64.8 Sex: 4 M, 0 F Diagnosis: NR Time since stroke: two early subacute < 3mos; two Chronic > 6mos	Int1: AO Int2: Synchronous AO + MI	EMG	A significant difference of the muscle strength between AO and AO + MI.
(91)	A-B-A reversal single experimental design	Inpatient	*n* = 3 Age (y): 50 ± 6.6 Sex: 1 M, 2 F Diagnosis: 3 I, 0 H Time since stroke: 25.3 mos	Int1: Asynchronous AO + MI with physical practice	3-D motion analysis MAL EMG	Occupational performance improved in all 3 subjects when applying AO + MI. All subjects showed improvement of motor functions.
(96)*	Single-group, within-subjects design with multiple assessment points	Inpatient	*n* = 2 Age (y): 53 Sex: 2 M Diagnosis: NR Time since stroke: 8 mos	Int1: Synchronous AO + MI Int2: Asynchronous AO + MI	EEG NHPT PST	The results of ERD, NHPT and PST are improved more effectively in synchronous AO + MI than asynchronous AO + MI.
(90)	A Graeco-Latin Square design. Single group pre-, post- and retention test.	Outpatient	*n* = 10 Age (y): 64.4 ± 9.4 Sex: 6 M, 4 F Diagnosis: 3 H, 7 I Time since stroke: 44.8 ± 15.98 mos	Int1: AO Int2: MI Int3: Synchronous AO + MI	Movement execution times ARAT SIS MIQ-3	Movement execution times decreased significantly in both the post-test and retention test compared to baseline. During retention, AO + MI resulted in significantly shorter execution times compared to both MI alone and the Control condition. Participants also reported clinically significant improvements in quality of life (SIS) and positive experiences with AO + MI.
(94)	quasi-experimental pre- and post-test control group study	Inpatient	*n* = 51 Age (y): 66.8 ± 14.1 Sex: 29 M, 22 F Diagnosis: 8 H, 43 I Time since stroke: 14.9 ± 5.5 days	EG: Synchronous AO + MI with usual care CG: Usual care	FMA-UE BBT KVIQ-10	No statistically significant differences in upper extremity motor function between the two groups. Subgroup analysis of the intervention group identified statistically significant (FMA-UE: *p* < 0.001; BBT: *p* = 0.04).

Intervention characteristics					
Study	Type of intervention	Intervention tasks	Session duration (mins)	Total number of sessions	Intervention time and frequency
(53)*	Int1: MI	Shooting basketball	30	1	1 session/day (1 day)
	Int2: AO	Shooting basketball	30	1	1 session/day (1 day)
	Int3: Synchronous AO + MI	Shooting basketball	30	1	1 session/day (1 day)
(52)	Int1: AO	Shooting basketball	NR	1	5 times per session (1 day)
	Int2: Synchronous AO + MI	Shooting basketball	NR	1	5 times per session (1 day)
(91)	Asynchronous AO + MI with physical practice	Beverage-drinking	NR	20	NR
(96)*	Int1: Synchronous AO + MI	Inserting and removing pegs	1.7	58	Everyday within 4 weeks
	Int2: Asynchronous AO + MI	Inserting and removing pegs	1.7	58	Everyday within 4 weeks
(90)	Int1: AO	Cup-stacking tasks	NR	48	1 session per week (5 weeks)
	Int2: MI	Cup-stacking tasks	NR	48	1 session per week (5 weeks)
	Int3: Synchronous AO + MI	Cup-stacking tasks	NR	48	1 session per week (5 weeks)
(94)	EG: Synchronous AO + MI with usual care	Twelve functional activities of the upper extremity with multiple repetitions displayed for each activity, included lighting a candle, stirring a pot of oatmeal, flipping playing cards, cleaning a counter, dispensing tape, scooping sugar, clicking a ball-point pen, retrieving tissues from a tissue box, rolling dice, retrieving a coffee cup, turning pages in a book, and pressing a button on a remote control.	25	6	3 times/week The length of their inpatient stay (average 2 weeks)
	CG: Usual care	NR	180	10	5 days a week The length of their inpatient stay (average 2 weeks)

Values are mean ± SD; An asterisk indicates the study includes neuroimaging data. EG indicates experiment group; CG, control group; Int, Intervention; NR, Not reported; F, female; M, male; mos, months; y, year; I, ischemia; H, hemorrhage; mins, minutes; AO, action observation; MI, motor imagery; AO + MI, Combined action observation and motor imagery; FMA-UE, Fugl-Meyer Assessment Upper Extremity; EMG, Electromyography; 3-D,3-dimensional; EEG, electroencephalogram; ERD, event-related desynchronization; MAL, Motor Activity Log; NHPT, Nine Hole Peg Test; PST, Pinch Strength Test; ARAT, action research arm test; SIS, Stroke Impact Scale; MIQ-3, Movement Imagery Questionnaire-3; BBT, Box and Block Test; KVIQ-10, Kinesthetic and Visual Imagery Questionnaire Short Version.

#### 3.1.2 Neurophysiological measure–neuroimaging outcomes

Nine studies (*n* = 130) were analyzed narratively for neuroimaging outcomes, comprising three studies that reported only neuroimaging results [see Table 3, (92, 93, 95)], four RCTs that reported both upper limb function and neuroimaging outcomes [indicated by an asterisk in Table 1 (49, 51, 54, 69)], and two non-RCTs that also reported both outcomes (indicated by an asterisk in Table 2 (53, 96). The neurophysiological measurements in these studies varied, with six studies using EEG (51, 53, 54, 93, 95, 96), one employing fNIRS (92), one using TMS (49), and one incorporating fMRI (69).

**Table 3:** Neuroimaging studies.

Study characteristics						
Study	Study design	Setting	Participants	Type of intervention	Outcome measure	Results
(95)	single group, within-subjects design	Outpatient	*n* = 10 Age (y): 60 ± 7.53 Sex: 9 M, 1 F Diagnosis: 3 H, 7 I Time since stroke: 57.5 ± 55 mos	Int1: Synchronous AO + MI with BCI Int2: MI with BCI	EEG	The ERD values and classification accuracy in AOMI were significantly greater than those under MI conditions.
(92)	single group, within-subjects design	Inpatient	*n* = 10 Age (y): 62.6 ± 12.0 Sex: 4 M, 6 F Diagnosis: NR Time since stroke: 1 mos	Int1: MI Int2: Synchronous other hand AO + MI Int3: Synchronous own hand AO + MI	NIRS VAS	MI vividness was significantly higher in synchronous own hand AO + MI intervention compared to the other conditions (*p* < 0.01). The activity of the cortical regions was also significantly enhanced (*p* < 0.01).
(93)	Between-subjects, cross-sectional design	Inpatient	*n* = 8 Age (y): 66 Sex: 6 M, 2 F Diagnosis: NR Time since stroke: NR	Int1: Gaze (AO) Int2: MI Int3: Synchronous AO + MI	EEG	The ERD of AO + MI was stronger than that of AO (*p* = 0.0002) or MI (*p* = 0.0091).

Intervention characteristics					
Study	Type of intervention	Intervention tasks	Session duration (mins)	Total number of sessions	Intervention time and frequency
(95)	Int1: Synchronous AO + MI with BCI	Wrist and hand extensions	7	2	1 session/day 2 days
	Int2: MI with BCI	Wrist and hand extensions	7	2	1 session/day 2 days
(92)	Int1: MI	Grasping a cup on a desk	15.15	1	1 session/day 1 day
	Int2: Synchronous other hand AO + MI	Grasping a cup on a desk	15.15	1	1 session/day 1 day
	Int3: Synchronous own hand AO + MI	Grasping a cup on a desk	15.15	1	1 session/day 1 day
(93)	Int1: Gaze (AO)	Hand opening and closing	3	1	1 session/day 1 day
	Int2: MI	Hand opening and closing	3	1	1 session/day 1 day
	Int3: Synchronous AO + MI	Hand opening and closing	3	1	1 session/day 1 day

Values are mean ± SD; Abbreviations: NR, Not reported; F, female; M, male; mos, months; y, year; I, ischemia; H, hemorrhage; mins, minutes; Int, Intervention; AO, action observation; MI, motor imagery; AO+MI = Combined action observation and motor imagery; BCI, Brain–computer interface; EEG, electroencephalogram; ERD, event-related desynchronization; NIRS, Near-infrared spectroscopy; VAS, Visual Analog Scale.

### 3.2 Study quality

**Figure 2:**
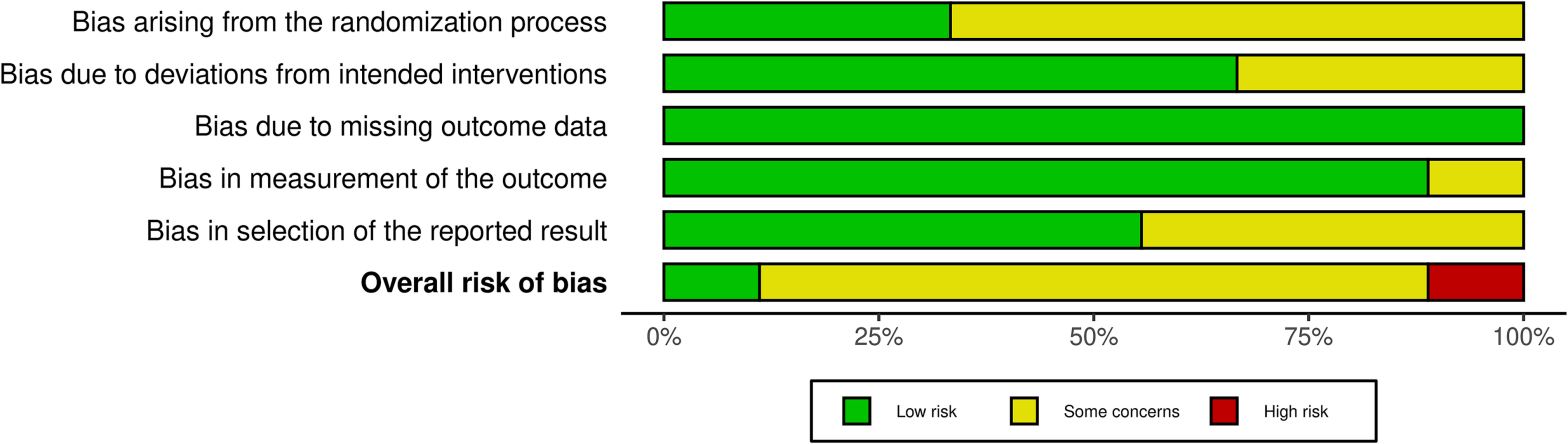
ROB2 summary. Summary of Risk of Bias 2 (ROB2) assessments, showing methodological quality across domains: randomization, adherence to interventions, handling of missing data, outcome measurement, and selection of reported results.

**Figure 3:**
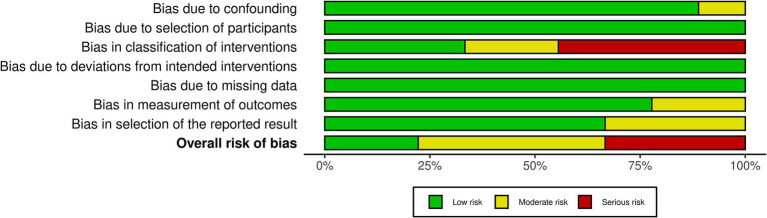
ROBINS-I summary. Summary of the ROBINS-I (Risk of bias in non-randomized studies of interventions) assessment, showing risk of bias across key domains: confounding, participant selection, intervention classification, missing data, outcome measurement, and selective reporting.

Among the nine included RCTs, seven were assessed to have some concerns of bias (51, 54, 64, 68, 69, 88, 89), and one was rated as having a high risk of bias (49), primarily due to issues with randomization and insufficient blinding (see Figure 2). Similarly, of the nine non-RCTs included, four were rated as having a moderate bias (52, 93, 94, 96) and three as having a high risk of bias (53, 92, 95), largely attributed to issues in the classification of interventions (see Figure 3). These limitations may have introduced bias in the reporting of subjective outcomes. Despite this, the GRADE assessment indicated that the overall quality of evidence for the FMA-UE and ARAT outcomes of upper limb motor function was moderate (see Table 4). The evidence was downgraded due to the high risk of bias and imprecision in effect estimates. No serious concerns were identified however regarding consistency or directness. Consequently, while potential bias in individual studies warrants a degree of caution, the moderate quality of the evidence permits a reasonable level of confidence in the estimated true effect.

**Table 4:** GRADE quality of evidence assessment for outcomes included in meta-analyses.

Outcome	No of participants (Studies)	GRADE assessment
FMA-UE	189 participants (7 studies)	⊕ ⊕ ⊕Ο ► reduced by one for risk of bias ► reduced by one for imprecision
ARAT	50 participants (2 studies)	⊕ ⊕ ⊕Ο ► reduced by one for risk of bias ► reduced by one for imprecision

FMA-UE, Fugl-Meyer Assessment Upper Extremity; ARAT, action research arm test.

### 3.3 Meta-analysis of RCTs investigating AO + MI practice effects on upper limb function

#### 3.3.1 FMA-UE outcomes

**Figure 4:**
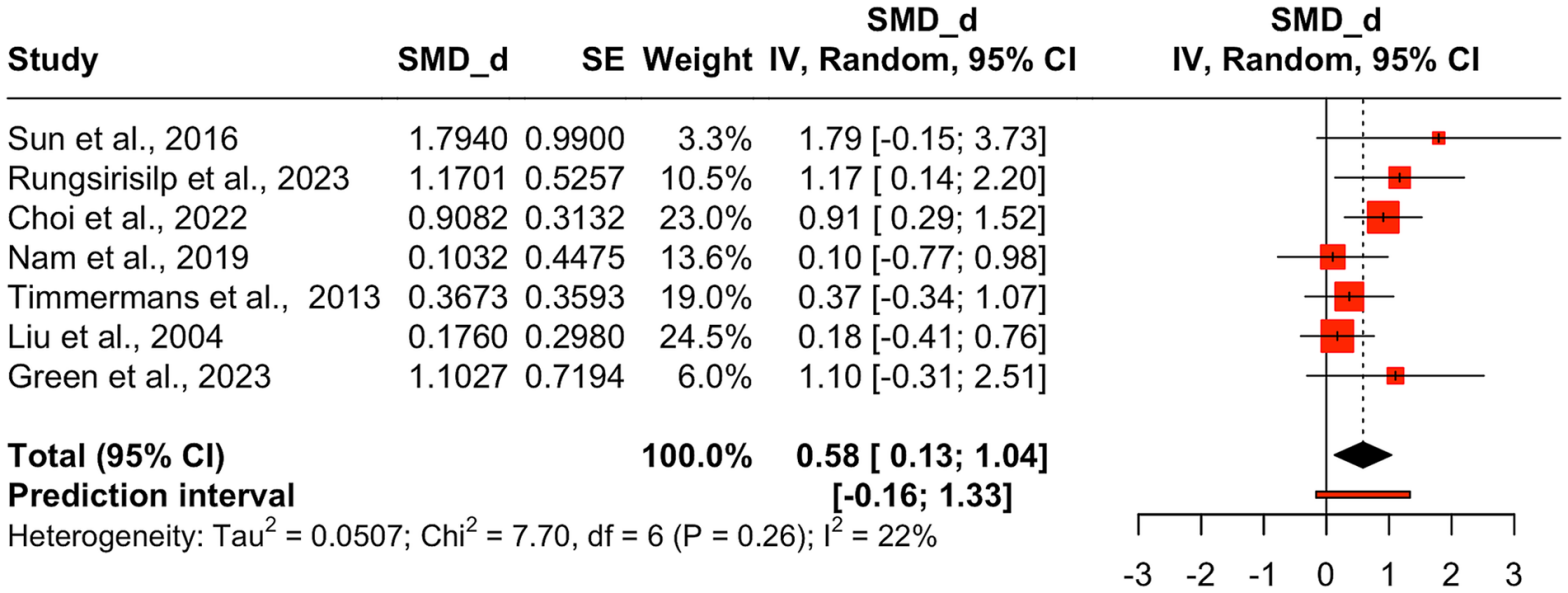
Pooled mean treatment effects of AO + MI interventions on FMA-UE total score. Pooled mean treatment effects of AO + MI (Action Observation and Motor Imagery) interventions on the FMA-UE (Fugl-Meyer Assessment for Upper Extremity) total score, based on results from multiple studies. Standardized mean differences (Cohen’s *d*, SMD_d) between intervention and control groups are presented with confidence intervals indicating precision.

Meta-analysis of the FMA-UE data incorporated seven effect sizes from seven studies to evaluate the overall impact of AO + MI practice on upper limb function. AO + MI practice showed a moderate and statistically significant improvement in upper limb function compared to control groups (*d* = 0.58, 95%CI: 0.13–1.04, *p* = 0.02). The between-study heterogeneity variance was estimated at *τ* = 0.23 (95%CI: 0.00–1.17), with I^2^ value of 22%. The prediction interval ranged from −0.16 to 1.33, suggesting a 95% chance that the effect size of new studies will fall within this range (see Figure 4).

#### 3.3.2 Combined FMA-UE and ARAT outcomes

**Figure 5:**
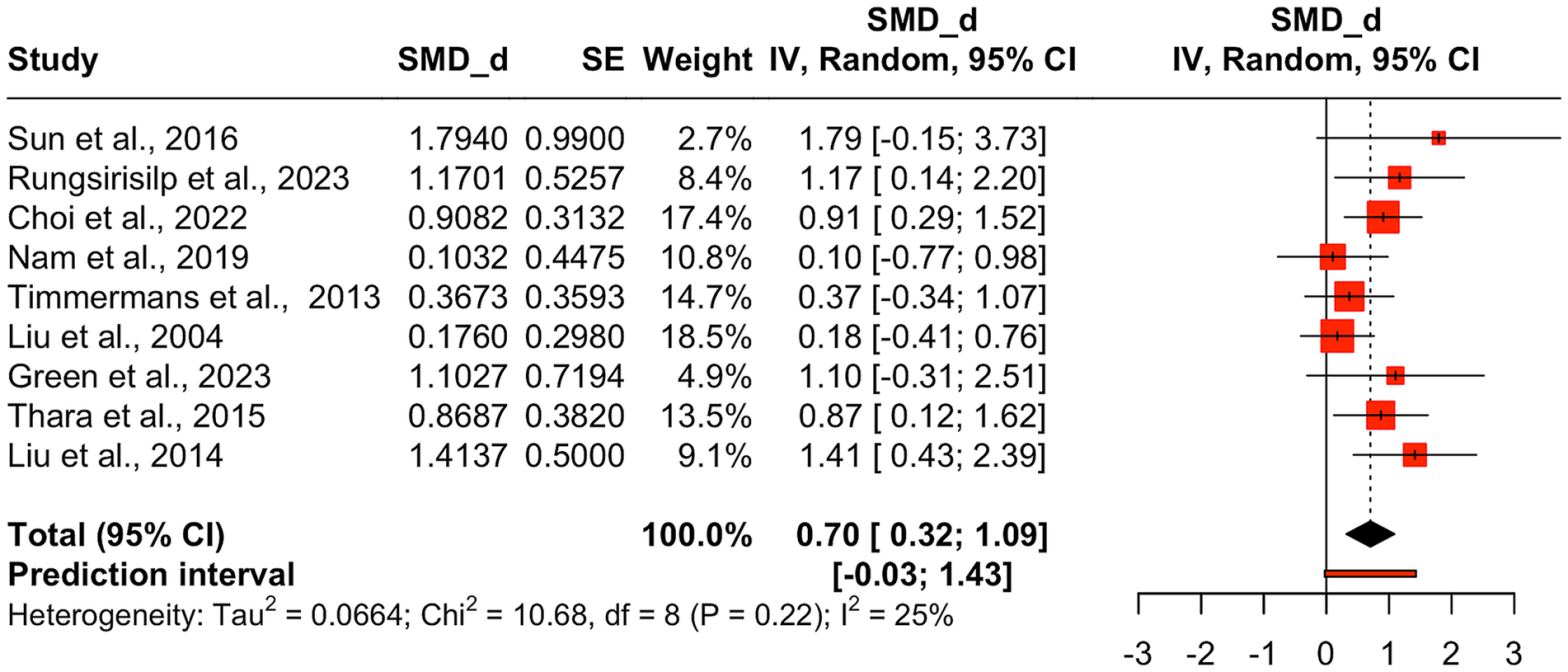
Pooled mean treatment effects of AO + MI interventions on combined FMA-UE and ARAT score. Pooled mean treatment effects of AO + MI (Action Observation and Motor Imagery) interventions on combined FMA-UE (Fugl-Meyer Assessment for Upper Extremity) and ARAT (Action Research Arm Test) total scores. Standardized mean differences (SMD, Cohen’s *d*) with confidence intervals summarize the impact of AO + MI interventions on upper limb motor function across multiple studies.

Given the limited number of ARAT studies [*n* = 2; (69, 72)], a secondary meta-analysis was conducted that included both FMA-UE and ARAT data. This approach to combine data from the two measures was adopted in favor of running a separate meta-analysis on the two studies using the ARAT measure. Previous research has recommended including a minimum of five studies for a meaningful meta-analysis (97, 98). While it is theoretically possible to meta-analyze just two studies, statistical inference is limited with such a small sample, and the added value is minimal because little new insight is gained beyond what the original studies already provide (97). The combined meta-analysis encompassed nine effect sizes from nine RCTs. AO + MI practice showed significant improvement in upper limb function, with results consistent with the initial analysis, but with a larger effect size (*d* = 0.70, 95%CI: 0.32–1.09, *p* = 0.003). The between-study heterogeneity variance was estimated at *τ* = 0.26 (95%CI: 0.00–0.92), with I2 value of 25%. The prediction interval ranged from −0.03 to 1.43, suggesting a 95% chance that the effect size of new studies will fall within this range (see Figure 5).

### 3.4 Meta-regression of moderating factors affecting upper limb motor function

A meta-regression analysis was conducted on all nine RCTs that were included in the second meta-analysis. This was to evaluate the influence of several moderating variables on AO + MI practice effects, in comparison to the overall upper limb function aggregate data included in the meta-analysis. These moderators included the time since stroke, age, intervention duration, arrangement of AO + MI (synchronous or asynchronous), BCI studies, control condition, and outcome measure.

Meta-regression analyses revealed no significant correlations between the examined moderators and the true effect size, indicating a robust effect of AO + MI on upper limb function in stroke patients. Specifically, the time elapsed since the stroke onset was not a significant moderator (*β* = 0.13, *p* > 0.05). Additionally, a non-significant negative correlation was identified between participants’ age and effectiveness (*β* = −0.04, *p* > 0.05), indicating that for each additional year of age, the effect size decreased by 0.04. Conversely, a non-significant positive relationship was found between intervention duration and effectiveness (*β* = 0.05, *p* > 0.05), showing that for each additional week of intervention, the effect size increased by 0.05.

**Figure 6:**
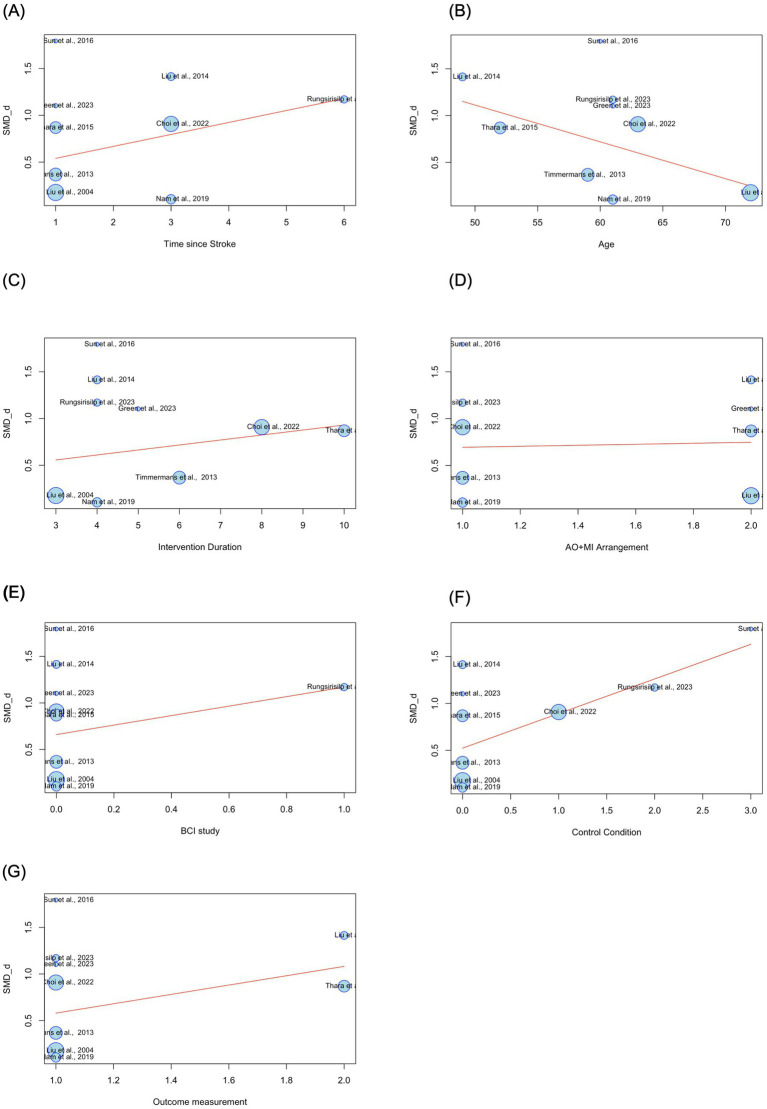
Estimated regression slopes and effect sizes in AO + MI interventions for upper limb function. Bubble size represents study weight, with larger bubbles representing studies with greater influence in the analysis. SMD_d = Cohen’s *d*. **(A)** 1 = early subacute (7 days to 3 months after stroke), 3 = late subacute (3–6 months after stroke), 6 = chronic (6 months or more after stroke). **(B)** Age is measured in years. **(C)** Intervention duration is measured in weeks. **(D)** 1 = synchronous AO + MI, 2 = asynchronous AO + MI. **(E)** 0 = non-BCI study, 1 = BCI study. **(F)** 0 = conventional therapy, 1 = AO, 2 = MI, 3 = asynchronous AO + MI. **(G)** 1 = FMA_UE, 2 = ARAT.

The arrangement of AO + MI (synchronous vs. asynchronous AO + MI) did not serve as a significant moderator (*β* = 0.05, *p* > 0.05). Furthermore, the inclusion of BCI technology in the interventions did not correlate with effect sizes (*β* = 0.51, *p* > 0.05). The control condition (comprising conventional therapy, AO, MI, and asynchronous AO + MI) also emerged as a non-significant moderator (*β* = 0.37, *p* > 0.05). Lastly, the outcome measure (FMA-UE and ARAT) did not serve as a significant moderator (*β* = 0.50, *p* > 0.05) (see Table 5). A bubble plot was used to visually represent the estimated regression slope and the effect size for each study included in the meta-regression analysis (see Figure 6).

**Table 5:** Influence of moderators on true effect size in AO + MI interventions for upper limb function.

Moderator	*N*	𝜷	*P*	Sig.	τ^2^	*I* ^2^
Time since stroke	9	0.13	0.28	No	0.03	13%
Age	9	−0.04	0.08	No	0.00	0%
Intervention duration	9	0.05	0.48	No	0.06	23%
AO + MI arrangement	9	0.05	0.89	No	0.11	36%
BCI study	9	0.51	0.43	No	0.07	27%
Control condition	9	0.37	0.11	No	0.01	3%
Outcome measure	9	0.50	0.23	No	0.04	18%

### 3.5 Sensitivity analyses

A sensitivity analysis was conducted by re-evaluating the two meta-analyses using previously calculated Hedge’s *g* values to mitigate concerns regarding small sample size bias. The results indicated no meaningful differences in the effect size estimates for the primary comparisons. Specifically, the meta-analysis of FMA-UE yielded a Hedge’s g value of 0.59 (95% CI: 0.14–1.04, *p* = 0.02), with an *I*^2^ value of 24%, while the meta-analysis of combined FMA-UE and ARAT outcomes produced a Hedge’s *g* value of 0.69 (95% CI: 0.32–1.06, *p* = 0.003), with an *I*^2^ value of 24%.

### 3.6 Small study effects

**Figure 7:**
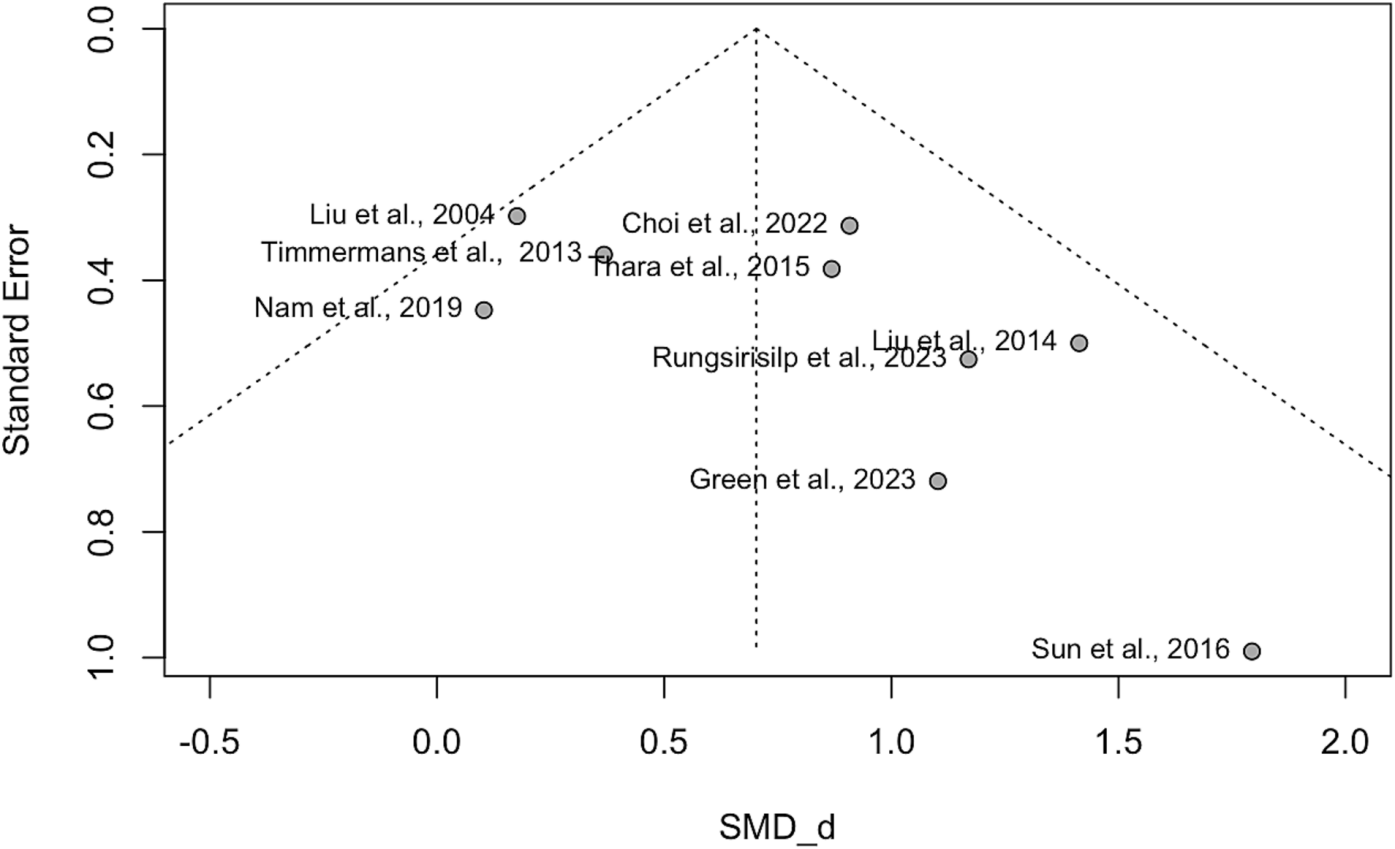
Funnel plot. This funnel plot visualizes the effect sizes of studies against their precision (standard error) in a meta-analysis. The *x*-axis represents the standardized mean differences (Cohen’s *d*, SMD_d = Cohen’s *d*), while the *y*-axis shows the precision of each study (inverse of standard error). A symmetric funnel indicates no publication bias, while asymmetry may suggest potential publication bias or heterogeneity among studies.

Small study effects (publication bias) were assessed across the nine included studies for FMA-UE and ARAT to evaluate the reliability of the results. The funnel plot revealed asymmetry, suggesting the potential presence of publication bias (see Figure 7). This observation was further examined using Egger’s test, which showed a positive intercept (b = 2.03), indicating some degree of asymmetry consistent with the funnel plot. However, the nonsignificant *p*-value (*p* = 0.10) suggests that the observed asymmetry is not statistically significant and could have arisen by chance. While this suggests some bias in the published literature, it is not strong enough to reach statistical significance. Further investigation with a larger sample size may be needed to determine the presence and extent of publication bias definitively.

### 3.7 Narrative review of non-RCTs investigating AO + MI practice on upper limb function

A total of six non-RCTs investigated the effects of AO + MI practice on upper limb function outcomes in stroke survivors (52, 53, 90, 91, 94, 96). Outcome measures across these studies included FMA-UE, grip strength, the 9-HPT, cup-stacking speed and EMG.

Two studies (90, 94) found no significant differences in clinical measures of upper limb function for AO + MI practice compared to AO, MI, or usual care. Both studies identified, however, that the improvements in upper limb function met the threshold for Minimal Clinically Important Difference (MCID) in various sub-scales of the motor assessment tools (99). In addition to reporting beneficial gains in other non-clinical measures [e.g., cup-stacking speed, (90)], both studies recommended AO + MI therapy as a valuable adjunct for neurorehabilitation in chronic stroke survivors (90, 94).

Robinson-Bert and Woods (94) investigated the impact of synchronous AO + MI practice on upper limb motor recovery after stroke, comparing this to usual care in a non-randomized pre- and post-test control group study. Participants (*N* = 51) received either AO + MI sessions three times per week along with usual care or usual care alone for 5 days per week, with an average inpatient stay of 2 weeks. Upper limb function was assessed using the FMA-UE and BBT at admission and discharge. Results showed no statistically significant differences between groups in FMA-UE scores (*U* = 324, z = −0.02, *p* = 0.99) or BBT scores (*U* = 274, z = −0.96, *p* = 0.34), and the effect sizes (*r*) were small. However, a higher proportion of the intervention group met the MCID for motor recovery (40% vs. 26.9%). Subgroup analysis indicated statistically significant improvements in upper limb recovery among intervention participants with greater commitment to the intervention (FMA-UE: *p* < 0.001; BBT: *p* = 0.04).

Binks et al. (90) examined the effects of synchronous AO + MI practice on upper limb recovery in chronic stroke survivors using a within-participant design, compared to AO, MI and an unpracticed control condition (transfer test). Using a Graeco-Latin square design, 10 participants completed 16 trials in each of the four conditions (AO + MI, AO, MI, Control) counterbalanced across four different cup-stacking task sequences, once a week over 5 weeks. Completion times for each task were measured at baseline (week 1), post-test (week 6), and at a two-week retention test (week 8). The study found a significant effect of practice condition on movement execution times at the retention test, *F* (3,77) = 5.42, *p* < 0.01, ηp^2^ = 0.17. Participants in the AO + MI condition (*M* = 21.37, SD = 6.62) completed tasks significantly faster than in the MI [*M* = 23.47, SD = 12.89, *t*(87.2) = 3.08, *p* < 0.05] and Control conditions [*M* = 25.87, SD = 11.93, *t*(87.2) = 2.71, *p* < 0.05]. The AO condition (*M* = 25.01, SD = 6.84) did not significantly differ from other conditions, though it approached significance compared to MI [*t*(87.2) = 2.61, *p* = 0.051]. No other significant differences were observed across conditions.

A separate study compared the effects of synchronous vs. asynchronous AO + MI on the upper limb function in two participants (96). Participant 1 (P1) engaged in synchronous AO + MI, while Participant 2 (P2) underwent asynchronous AO + MI, both performing a peg insertion and removal task. The NHPT and PST were administered at baseline and after 1, 2, 3, and 4 weeks of intervention. After 4 weeks of consecutive training, both participants struggled to complete the tasks within the designated time limits. However, P1 was able to insert 6 pegs, while P2 managed to insert 4 pegs into the holes on the pegboard. Initially, P1 had a lower PST score than P2; nevertheless, P1 consistently achieved higher scores than P2 beginning in the second week of the intervention. Overall, the higher NHPT and PST scores following synchronous AO + MI indicated that P1 experienced greater improvements in motor function of the affected hand compared to P2.

Three small sample studies reported EMG outcomes and consistently found that AO + MI practice led to greater muscle strength and activity compared to either AO or MI individually (52, 53, 91).

Huang et al. (52) evaluated the feasibility of a VR-based MI training system combined with EMG-based real-time feedback to enable personalized training and quantify participation. A single-group study with four participants performed three sequential experiments on the same day: an assessment, AO, and synchronous AO + MI, each involving a bilateral upper limb basketball-shooting task. EMG data were collected from the flexor pollicis longus, flexor digitorum superficialis, and flexor carpi radialis muscles. Results indicated that muscle strength remained relatively stable in the AO experiment across participants, with muscle activation curves for AO closely matching those of the relaxed state, consistent with low activation levels during AO. In contrast, the synchronous AO + MI experiment showed greater average muscle strength and more dynamic changes in muscle activation, with variability observed between participants. This suggests that EMG-based feedback effectively assessed participant engagement during MI-based rehabilitation. Overall, real-time EMG feedback scores showed superior performance in the AO + MI condition compared to AO.

Lin et al. (53) investigated the effects of combining VR-based AO with EMG-based real-time feedback on MI training. In their single-group study, eight participants completed four sequential experiments over 4 days: an initial assessment, MI, AO, and synchronous AO + MI, all involving a bilateral upper limb basketball-shooting task. EMG signals were collected from seven channels of a passive electrode box placed on key upper limb muscles, including the flexor digitorum superficialis, flexor pollicis longus, and flexor carpi radialis in both arms. The flexor digitorum superficialis on each arm served as its own reference, while the intermediate clavicle muscle acted as the ground electrode. Results showed that AO produced steady, low muscle strength values similar to those in the relaxed state. Both MI and AO + MI produced higher and more variable muscle strength values, with AO + MI showing the highest levels, indicating that AO can enhance MI training. Overall, AO + MI led to superior performance compared to MI alone, yielding greater muscle strength and basketball-shooting scores.

Cha et al. (91) investigated the impact of AO + MI practice on EMG outcomes, occupational performance and motor function using an A-B-A study design with follow-up. Three participants completed 20 asynchronous AO + MI sessions using a beverage-drinking task, with 3-dimensional (3D) motion analysis and EMG data recorded during each session. Assessments using the MAL and Assessment of Motor and Process Skills (AMPS) were conducted in pre- and post-intervention phases (after 20 sessions) and at a 2-week follow-up. Post-treatment results indicated significant improvements across participants. 3D motion analysis revealed enhanced movement smoothness for all, with increased elbow movement range noted only in participant 1. EMG analyses showed elevated agonist muscle activity (%RVC) in reaching movements, with changes of 93.3, 28.8, and 58.2% for participants 1, 2, and 3, respectively, and reductions in co-contraction ratios (CCR) of 224.7, 234.9, and 221.3%, suggesting improved coordination. Additionally, MAL scores indicated sustained enhancements in the use and quality of the affected side, with participant 1 achieving statistically significant improvements in both use (>2.0) and AMPS motor skills (>0.5 logit) at follow-up.

### 3.8 Narrative review of AO + MI practice on neurophysiological outcomes

Nine studies, comprising five non-RCTs (53, 92, 93, 95, 96) and four RCTs (49, 51, 54, 69), examined the effects of AO + MI practice on neuroimaging outcomes in stroke survivors, including EEG, fNIRS, TMS and fMRI.

#### 3.8.1 EEG studies

Six studies, consisting of four non-RCTs (53, 93, 95, 96) and two RCTs (51, 54) employed EEG to assess the effect of synchronous AO + MI on ERD recorded over the primary motor cortex. All studies reported significantly greater ERD values in the synchronous AO + MI group compared to control groups (which included AO, MI, and asynchronous AO + MI).

Ichidi et al. (93) analyzed EEG signals from stroke patients (n = 8) and healthy participants (n = 8) during three tasks conducted in a single session: gazing at a static image (Gaze), MI, and AO + MI, all involving a hand opening and closing task. ERD was analyzed to compare the responses between stroke patients and healthy subjects, and among the three tasks. For the alpha band, a significant main effect of task was observed, *F* (1.29,15.48) = 3.996, *p* = 0.055, but *post hoc* tests indicated no significant differences in ERD amplitude among the Gaze, MI, and AO + MI conditions. In the beta band, significant main effects were found for both group and task. The group effect, *F* (1,12) = 5.354, *p* = 0.039, showed that stroke patients exhibited greater ERD amplitudes than healthy subjects. The task effect, *F* (2,24) = 15.697, *p* < 0.001, indicated that ERD for AO + MI was significantly stronger than for both Gaze (*p* = 0.0002) and MI (*p* = 0.0091), with MI also being stronger than Gaze (*p* = 0.023). No other effects were significant (*p* > 0.1).

Lin et al. (53) examined the impact of integrating VR-based AO with EMG-based real-time feedback on MI training in a single-group study involving eight participants. Over 4 days, participants completed four sequential tasks: an initial assessment, MI, AO, and synchronous AO + MI, all related to a bilateral upper limb basketball-shooting task. EEG signals were recorded from 18 channels centered on C3 and C4 in the sensorimotor cortex. The analysis focused on alpha and beta band power across the four tasks. Both the initial assessment and MI conditions displayed similar patterns, with alpha power exceeding beta power in the sensorimotor cortex. Additionally, beta activity showed greater variability and higher power when comparing MI to AO and AO + MI. Notably, alpha power in the AO + MI condition was lower than that of the beta band, while the alpha trend in AO aligned with both the initial assessment and MI. A comparative analysis using the non-parametric Friedman’s test assessed changes in power across frequency bands, averaging and normalizing electrode data for each condition. Both the initial assessment and MI exhibited similar power ranges in the alpha and beta bands, consistent with spectrum power maps. AO + MI revealed a distinct decrease in normalized power response compared to other conditions, likely due to the combined effects of MI training and the enhancing influence of AO on ERD in the alpha band. In contrast, the beta frequency analysis indicated significant differences in AO compared to initial assessment and MI. Unlike alpha power, beta power increased in both AO and AO + MI conditions, suggesting that observing movement in a virtual environment enhanced beta activity. However, AO + MI demonstrated lower beta power than AO, potentially reflecting the influence of MI.

Sun et al. (51) examined the impact of synchronous versus asynchronous AO + MI practice on sensorimotor cortex activation during a peg insertion and removal task. Participants were randomly assigned to either a synchronous AO + MI experimental group (EG, *n* = 5) or an asynchronous AO + MI control group (CG, *n* = 5). Sensorimotor cortex excitability was assessed via ERD data recorded over the primary motor cortex (i.e., mu rhythm suppression). Results indicated that synchronous AO + MI significantly enhanced sensorimotor cortex activation compared to asynchronous AO + MI (ERD: *F* = 12.800, *p* = 0.007) after 4 weeks of practice. By the study’s conclusion, the EG showed consistently higher mean ERD values than the CG, with effect sizes progressively increasing across sessions (W1: *d* = 0.684, W2: *d* = 1.518, W3: *d* = 2.143, W4: *d* = 2.532). Independent *t*-tests further confirmed that ERD differences between groups reached statistical significance (*p* < 0.05) after 2 weeks of practice.

Sun et al. (51) extended the work of Sun et al. (96), which compared synchronous and asynchronous AO + MI effects on upper limb function in two participants performing a peg insertion and removal task. Participant 1 (P1) practiced synchronous AO + MI, while Participant 2 (P2) practiced asynchronous AO + MI. EEG was recorded over the right-hand motor cortex (C3, FC3, CP3, T7, Cz) to assess sensorimotor rhythm (SMR) ERD during MI. At baseline, the cortical excitation levels during MI were equivalent between participants. Following MI practice, both participants showed enhanced ERD in SMR. However, after 4 weeks of MI practice, P1 displayed an ERD pattern with greater amplitude, longer duration, and involvement of additional frequency components compared to P2.

Rungsirisilp and Wongsawat (95) examined the effects of an AO + MI-based BCI on ERD and classification performance in chronic stroke patients. Using a within-subjects repeated measures design, 10 participants performed both an MI task as the control condition and an AO + MI task as the experimental condition across two sessions. For the MI task, participants performed MI while viewing a static arrow image, while for the AO + MI task, they observed a video of the movement while engaging in MI of this action. Cortical activity in the AO + MI and MI conditions was compared using ERD/ERS and time-frequency analyses, revealing significant differences in ERD/ERS values in the alpha (*p* = 0.005), lower beta (*p* = 0.013), upper beta (*p* = 0.022), and full frequency bands (*p* = 0.007). Similarly, classification performance in the alpha (*p* = 0.005), lower beta (*p* = 0.011), upper beta (*p* = 0.021), and full bands (*p* = 0.005) was significantly higher in the AO + MI condition compared to the MI condition.

Rungsirisilp et al. (54) extended this work by comparing the effects of AO + MI-based BCI and MI-based BCI on cortical excitation and cognitive task performance. Seventeen stroke participants were randomly assigned to either an AO + MI-based BCI group (*n* = 9) or an MI-based BCI group (*n* = 8). Cortical excitation in the affected sensorimotor hand region was assessed using ERD, while cognitive task performance was measured through online classification accuracy. Following 40 AO + MI or MI trials, EEG data were analyzed from five channels over the affected sensorimotor region (FC3, C5, C3, C1, CP3 for right-sided hemiparesis; FC4, C6, C4, C2, CP4 for left-sided hemiparesis). Results showed that the AO + MI-based BCI group had significantly higher mean ERD in the alpha and beta bands and greater classification accuracy than the MI-based BCI group (*p* = 0.034, 0.021, and 0.038, respectively). However, both groups showed similar, progressive increases in classification accuracy over time.

#### 3.8.2 fNIRS study

In the study by Fujiwara et al. (92), fNIRS was used to measure cortical activity in 10 stroke patients under three conditions: MI, synchronous AO + MI using the other hand, and synchronous AO + MI using the patient’s own hand. Each condition involved a single session where participants imagined grasping a cup on a desk, with conditions performed in random order. Recordings were obtained over the left and right sensorimotor cortices, premotor area, prefrontal cortex, presupplementary motor area, and supplementary motor area. Results indicated that cortical activity increased compared to baseline across all regions in all three MI conditions, while no interaction effects were observed. Notably, oxygenated hemoglobin levels (Z-score) were significantly higher in the own-hand AO + MI condition than in the MI or other-hand AO + MI conditions [*F* (1,2) = 37.327, *p* < 0.01].

#### 3.8.3 TMS and fMRI studies

Choi et al.’s (49) RCT investigated the effects of AO + MI practice on corticospinal excitability using MEP amplitude as the main outcome measure. Forty-five stroke patients were randomly assigned to either an experimental group receiving synchronous AO + MI (*n* = 22) or a control group undergoing AO (*n* = 23). Both groups engaged in 10 activities of daily living (e.g., using chopsticks, writing with a pen, and hand washing), from which participants selected five meaningful activities to practice for five times a week over an 8-week training period. Pre- and post-intervention evaluations indicated significant changes in MEP amplitude within both the experimental and control groups. While the between group comparisons were not significant, there was a significant increase in MEP amplitude observed in the experimental group only, when comparing the amount of change before and after the intervention (*p* = 0.001).

In the study by Liu et al. (69), fMRI was employed to investigate the effects of combining mental practice with physical practice on hand function in stroke patients. Twenty stroke patients were randomly assigned to an experimental group receiving asynchronous AO + MI alongside physical practice (*n* = 10) or a control group who received physical practice alone (*n* = 10). In the experimental group, participants observed a video before engaging in physical practice followed by MI (asynchronous AO + MI). The tasks involved flexion and extension of the thumb, abduction and adduction of all fingers, making a fist, spreading the hand, moving extended fingers back and forth, and performing ulnar and radial deviations. Hand function was assessed using the ARAT, while the number of activated voxels in the contralateral somatosensory motor cortex (SMC) was measured via fMRI.

Results indicated significant increases in ARAT scores post-training for both groups (*p* < 0.01), with greater improvements observed in the experimental group compared to the control group (*p* < 0.05). Comparing the number of activated voxels in the contralateral SMC before and after training revealed a significant main effect of training [*F*(1,154) = 9.558, *p* < 0.01], indicating that the number of activated voxels post-training was greater than pre-training. There was also a significant main effect of group [*F*(1,75) = 4.629, *p* = 0.035], with the treatment group showing a greater increase in the number of activated voxels compared to the control group. Furthermore, the number of activated voxels in the contralateral SMC and the change in ARAT scores were positively correlated with the use of MI involving the affected hand in the treatment group.

## 4 Discussion

This systematic review with meta-analysis is the first to evaluate the effectiveness of AO + MI practice on upper limb function and neurophysiological outcomes in individuals with stroke. Our study also investigated the moderating effects of demographic and clinical factors that could potentially influence treatment outcomes. Overall, this review identified AO + MI practice can significantly enhance clinical measures of upper limb recovery compared to control groups, this indicates that AO + MI practice represents a potentially effective tool for stroke rehabilitation. In the data pooled across nine RCT studies, there were no significant effect of moderating variables, indicating robust AO + MI practice effects across sub-populations and arrangements of AO + MI. Our narrative synthesis identified a dominant effect for AO + MI practice compared to comparators across behavioral and neurophysiological outcomes, although several studies reported small sample sizes. Below we discuss the main findings.

### 4.1 AO + MI practice on upper limb motor function outcomes

In both meta-analyses, one using FMA-UE scores only and the other combining FMA-UE and ARAT scores, AO + MI practice demonstrated a medium positive overall effect on upper limb function compared to control conditions. These findings demonstrate the potential of AO + MI interventions to enhance upper limb function in stroke patients, which supports the proposals in previous narrative review papers (16, 18, 46). Although FMA-UE and ARAT assess different constructs within the ICF framework (body function vs. activity), our meta-regression did not indicate a significant moderating effect of outcome type. Therefore, we report both a focused FMA-UE analysis and a combined analysis to provide a broader view of intervention effects, while acknowledging the conceptual differences and small number of ARAT studies. In the present study, the 95% confidence interval (CI) for the pooled effect size (0.13 to 1.04) indicates a reliable increase in FMA-UE scores for the intervention group, with the upper bound of the CI (1.04) suggesting a potentially substantial improvement in upper limb function. This finding is consistent with trends in individual studies that also demonstrated improvements in FMA-UE outcomes following the intervention. Furthermore, the mean improvement of 5.8 points for AO + MI in the FMA-UE data is within the MCID threshold of approximately 4–12 points established for stroke patients (100). It is important to acknowledge that portions of the CI do fall below this threshold, indicating that the effects may not always be clinically meaningful. Despite this, improvements in upper limb function were consistently observed across the studies reviewed, suggesting that AO + MI could serve as a valuable adjunct to conventional stroke rehabilitation therapies.

The meta-analyses exhibited low heterogeneity (<40%), reinforcing the reliability of our results. Additionally, the prediction interval indicated a positive trend in upper limb function improvement, ranging from −0.29 to 1.39. This range suggests a 95% probability that the effect size of future studies will fall within these bounds, with the potential for minimal negative effects at the lower end and substantial improvements at the upper end. Overall, this interval highlights the expected variability in treatment effects, suggesting that future interventions are likely to continue to yield positive outcomes for upper limb rehabilitation.

Similarly, the findings from six non-RCTs collectively indicate that AO + MI practice offers potential benefits in upper limb rehabilitation (52, 53, 90, 91, 94, 96). Notably, three EMG studies reported that AO + MI practice resulted in superior muscle strength and activity compared to either AO or MI (52, 53, 91). This indicates that AO + MI practice may not only improve motor function but also enhance the activities of daily living, for example with potential improvements in movement smoothness, muscle activation, and coordination in stroke survivors.

Huang et al. (52) and Lin et al. (53) explored the use of AO within a VR environment, demonstrating that the VR-based AO component could facilitate MI. Moreover, both studies demonstrated that EMG-based real-time feedback served as an effective method for monitoring patient engagement during MI tasks. A recent study found that combining VR-based MI with kinesthetic MI enhances brain rhythmic patterns and improves task differentiation compared to kinesthetic MI alone (101). Three behavioral studies recommended AO + MI practice as a valuable adjunct for neurorehabilitation in chronic stroke survivors (90, 94, 96). Overall, these studies reiterate the usefulness of AO + MI as an effective intervention for patients demonstrating commitment to their rehabilitation, particularly in facilitating upper limb recovery following stroke.

### 4.2 AO + MI practice on neuroimaging outcomes

The narrative review of neuroimaging results from nine studies, consisting of five non-RCTs (53, 92, 93, 95, 96) and four RCTs (49, 51, 54, 69), consistently demonstrated that AO + MI improved both cortical activation and upper limb function in stroke survivors. The neuroimaging techniques employed in these studies offers a multimodal understanding of how AO + MI practice can produce relatively permanent changes in cortical activation that correspond to motor recovery. This captures various aspects of neural activity, particularly cortical excitation, thereby clarifying the mechanisms underlying motor recovery (102). The evidence from these studies aligns with substantial multimodal neuroimaging evidence showing similar effects in healthy adults, whereby AO + MI practice can facilitate corticomotor engagement of brain areas involved in the mentally practiced task (46, 103). The present systematic review of nine studies therefore extends the evidence base showing that AO + MI practice can enhance upper limb function by promoting cortical activation that supports neuroplastic changes that underpin recovery in stroke survivors.

In the six EEG studies, which included four non-RCTs (53, 93, 95, 96) and two RCTs (51, 54), greater ERD values were obtained in the synchronous AO + MI group relative to the comparison groups, which included AO, MI, and asynchronous AO + MI. The stronger ERD values observed in EEG studies indicate enhanced cortical engagement during synchronous AO + MI. This could potentially correspond to the neuroplasticity effects that are essential for recovery.

These findings for EEG in stroke survivors are consistent with a previous study that reported increased electrophysiological activity in the primary sensorimotor and parietal regions, particularly within the mu/alpha and beta frequency bands, during AO + MI compared to separate AO or MI conditions (45). Additionally, the studies conducted in stroke survivors by Rungsirisilp et al. (54) revealed that the AO + MI-based BCI group had significantly higher ERD values compared to the MI-based BCI group. This finding supports the conclusion that the enhanced performance of the AO + MI-based BCI group, relative to the MI-based BCI group, demonstrates that combining AO with MI can enhance the effectiveness of MI, promoting brain plasticity and functional recovery.

Notably, the fMRI study conducted by Liu et al. (69) reported that AO + MI combined with physical practice led to a stronger BOLD response in the contralateral sensorimotor cortex compared to physical practice alone. That study also identified the positive correlations between increased BOLD levels in the sensorimotor cortex and improved hand function, suggesting that AO + MI may effectively enhance motor recovery by facilitating neuroplasticity (69). This finding is consistent with recent research demonstrating that AO + MI practice can enhance the capacity for neuroplasticity in individuals who typically do not respond to intermittent theta burst stimulation, potentially facilitating neurological recovery (104).

Fujiwara et al.’s (92) fNIRS data highlighted the potential benefits of using inverted images of a stroke patient’s nonparalyzed hand during MI to enhance motor recovery, since own-hand AO + MI enhanced MI vividness and cortical activity during motor task execution. In line with this, MI vividness was correlated with corticospinal excitability during AO + MI (105). Those authors recorded MI vividness using a visual analogue scale, which they proposed as a potential proxy measure of corticospinal excitability, particularly in complex MI tasks. Additionally, research suggests that observing a high-performing individual with similar motor deficits may be more effective for recovery than observing an unimpaired model (106). A recent review further highlighted that similarities and differences between the observer and model, such as self-observation or sex differences, can influence these effects (107).

In summary, these studies support the view that AO + MI practice can increase and broaden activity within the motor execution network, and that these neurophysiological changes correspond to improved upper limb function in stroke survivors. These findings are consistent with proposals that AO + MI practice represents an effective complementary intervention for promoting upper limb motor recovery (18, 46). Furthermore, AO + MI is both cost-effective and accessible, making it a practical option for widespread clinical implementation (16).

### 4.3 Factors associated with improvement in upper limb motor function

The meta-regression analysis revealed no significant correlations between the examined moderators and the true effect size, suggesting that the overall effect of AO + MI on upper limb function in stroke patients is robust across various study conditions. Although none of the tested moderators reached statistical significance in the meta-regression, this does not rule out their potential clinical importance. The lack of significant moderation may be due to limited statistical power (e.g., small number of studies per subgroup), limited variability in moderator values, or unmeasured confounding factors. For example, the absence of a significant effect for BCI involvement or AO + MI arrangement may reflect the heterogeneity in how these elements were implemented across studies. Similarly, outcome measure (FMA-UE vs. ARAT) did not significantly influence the pooled effect size, suggesting that AO + MI may benefit both impairment and activity domains, though more studies using ARAT are needed.

Furthermore, the direction and magnitude of the relationships between each moderator and the effect size were examined through regression coefficients. This exploratory analysis identified potential trends that may merit further investigation. The regression coefficients for time since stroke, age, intervention duration, and arrangement of AO + MI were small, indicating a limited association between these factors and the observed effect size.

A small positive correlation was found between time since stroke and treatment effectiveness (*β* = 0.13), indicating that, in this study, treatment effects slightly increased with longer time since stroke. However, this finding contrasts with broader evidence indicating that the most substantial improvements occur early after stroke onset, followed by a gradual decline in improvement over time (83). Furthermore, limited induced motion interventions for upper limb rehabilitation within 2 weeks post-stroke has been shown to produce better outcomes than later interventions (108). Additionally, time since stroke may influence patterns of cerebral reorganization, as patients with poorer outcomes tend to recruit the contralesional middle intraparietal sulcus, contralesional cerebellum, and ipsilesional rostral premotor cortex primarily in the early post-stroke phase rather than in the later stages (85). Multiple factors influence stroke recovery, including urinary incontinence, sex, pre-stroke disability, dysarthria, age, dysphasia, and limb deficits (109); however, at least 16% of improvements in body function and activities can be attributed to time alone (83). The relationship between time since stroke and the effectiveness of AO + MI has not yet been explored, highlighting the need for further research.

Notably, a negative correlation was observed between participants’ age and treatment effectiveness (*β* = −0.04), indicating a decrease in effect size of 0.04 for each additional year of age among participants aged 49 to 72. This implies that AO + MI interventions may be slightly less effective in older stroke patients. This result aligns with Mulder et al.’s (84) study, which examined the relationship between age and imagery capacity. They assessed scores from the Vividness of Movement Imagery Questionnaire among 333 participants divided into three age groups: < 30 years, 30 to 64 years, and > 64 years. Their findings indicated that elderly participants demonstrated slightly lower MI capacity than younger participants, particularly in first-person MI (84). Therefore, the relationship between age and AO + MI practice requires further investigation.

In contrast, a positive relationship was identified between intervention duration and effectiveness (*β* = 0.05), indicating an increase in effect size of 0.05 for each additional week of AO + MI intervention, with a duration range of 3 to 10 weeks. This highlights the potential importance of longer treatment periods for achieving optimal upper limb recovery. This result is consistent with a Cochrane review that assessed the impact of variations in total rehabilitation time on stroke recovery regarding activity. Their findings suggested that increased time spent in rehabilitation may be beneficial if it exceeds a certain threshold (86). However, no current studies examine the relationship between intervention time and AO + MI practice.

The arrangement of AO + MI did not emerge as a significant moderator, as indicated by the minimal regression coefficient (*β* = 0.05). This indicates that the two forms of AO + MI (synchronous and asynchronous) do not have a substantial impact on the effectiveness of the intervention. Sun et al. (51) compared the effects of synchronous and asynchronous AO + MI on upper limb function in *N* = 10 stroke participants. Their results showed that synchronous AO + MI significantly enhanced sensorimotor cortex activation and upper limb function compared to asynchronous AO + MI after 4 weeks of practice. The authors concluded that synchronous AO + MI more effectively stimulates the sensorimotor cortex, promoting faster neurorehabilitation in stroke patients. While this small-sample study is the only one comparing these two modalities in stroke rehabilitation, it highlights a notable gap in the literature. While this picture is more mixed across studies in healthy adults (48), further research is necessary to examine the different impacts of synchronous and asynchronous AO + MI on stroke recovery.

The regression coefficients for studies incorporating BCI technology, control conditions (including conventional therapy, AO, MI, and asynchronous AO + MI), and outcome measures were notably larger compared to other factors, suggesting a moderate positive association between these variables and the effect size. Specifically, studies that utilized BCI techniques reported larger effect sizes (*β* = 0.51), indicating that the integration of BCI technology may enhance the effectiveness of AO + MI interventions (54). This finding is consistent with previous research that suggests BCIs can promote neuroplasticity by providing real-time feedback and active engagement in MI tasks, thereby facilitating more effective motor recovery (95).

A recent review found that most studies on robot control focus on initiating grasping or pinching movements through MI, often combining kinesthetic and visual feedback from the robotic device and display screen (110). While MI in BCI-hand robots show promise for stroke-affected hand rehabilitation, the evidence remains insufficient. Additionally, these studies displayed significant variability in reporting, highlighting the need for standardized protocols to assess technical and clinical outcomes, enhancing the evidence base for these interventions (110).

A similarly strong association was observed between the choice of outcome measure and the effect size (*β* = 0.50), with stronger effects reported for the ARAT (69, 72) compared to the FMA-UE. This emphasizes the need for consistency and rigor in the selection of assessment tools to accurately capture the benefits of AO + MI interventions. The variability in reported effect sizes across studies may be influenced by the sensitivity and specificity of the different assessment instruments employed. In this context, our review builds upon previous reviews that combined the FMA-UE and ARAT outcomes in a meta-analysis (25, 111). By integrating both FMA-UE and ARAT results into the combined FMA-UE and ARAT score meta-analysis to provide a more nuanced evaluation of the impact of AO + MI on upper limb function on separate indicators of arm function.

The FMA-UE and ARAT are two of the most widely used upper-extremity scales, as both show similar effectiveness in detecting changes in a patient’s upper-extremity function over time (112). Previous research has demonstrated a strong association between motor scores achieved on the FMA-UE and measured capacity on the ARAT, with significant overlap in their respective areas under the curve. Specifically, FMA-UE scores less than 31 points are indicative of no to poor arm-hand capacity (≤21 points) on the ARAT, while scores above 31 correspond to limited to full arm-hand capacity (≥22 points) on the ARAT (113). However, The FMA-UE and ARAT reflect different underlying constructs as defined by the International Classification of Functioning, Disability and Health (ICF) model, with the FMA-UE focusing on body function levels and the ARAT addressing activity capacity (113, 114).

The FMA-UE includes 33 items assessing the upper paretic limb, categorized into four subsections: shoulder-arm, wrist, hand, and upper limb coordination, with a maximum score of 66 points using a three-point ordinal scale (115). In contrast, the ARAT consists of 19 functional items divided into four subtests: grasp., pinch, grip, and gross motor function, with a maximum total score of 57 indicating normative performance (116). Consequently, standardizing the use of these outcome measures in future research could enhance the comparability and validity of findings, ultimately leading to more effective stroke rehabilitation interventions. Consistent use of validated assessment tools will improve our understanding of how AO + MI interventions facilitate motor recovery, paving the way for more tailored rehabilitation strategies.

The positive association with control conditions suggests that the type of control used (conventional therapy, AO, MI, mirror therapy or asynchronous AO + MI) can influence the magnitude of the observed effects. In this meta-analysis, we did not analyze AO + MI in comparison with each control type separately due to the high variability among control conditions in the included studies. Specifically, only one study used MI-based BCI as a control (54), two studies used AO (49, 69), one used mirror therapy (72), and four studies used conventional therapy (64, 68, 88, 89). This variation limits the scope to draw precise conclusions about the comparative effectiveness of AO + MI versus each control condition. Therefore, given the variability in control conditions across existing studies and the limited number of studies directly comparing AO + MI with AO and MI in stroke rehabilitation, further research is essential to systematically evaluate AO + MI relative to individual control conditions, such as AO, MI, and conventional therapy.

Notably, many included studies employed subtractive control designs, comparing AO + MI to either AO or MI alone. While this design is valuable for isolating the additive or synergistic effects of AO + MI, especially in studies using neurophysiological or neuroimaging methods, it may reduce between-group differences in functional outcomes. Since both groups receive active components of therapy, differences in treatment intensity or cognitive engagement are diminished relative to a no-treatment control, potentially underestimating the true effect size in meta-analyses. Future studies should consider incorporating both subtractive and passive control conditions to better assess both the mechanisms and clinical efficacy of AO + MI interventions.

In summary, by comprehensively considering these factors, future studies can reduce variability and achieve more consistent and reliable results. This highlights the need for standardized protocols that specify optimal intervention durations, to consider both the use of advanced technologies like BCI, VR and the selection of rigorous outcome measures when evaluating the impact of AO + MI interventions in stroke rehabilitation (122).

### 4.4 Limitations and future research recommendations

A significant challenge identified in this review is the inconsistent use of terminology across studies. Some studies claim to focus solely on AO or MI but in practice, include elements of both. Moreover, a wide range of terms was used to describe similar concepts, such as mental practice, motor imagery, synchronous AO + MI, asynchronous AO + MI, and combined AO + MI, leading to confusion and difficulties in comparing results across studies. For example, the range of terms used for AO + MI in the reviewed studies included AO combined MI, the mode of mental practice, mental imagery, mental practice, mental practice using inverse video, synchronous/asynchronous AO + MI, AOMI-based BCI, motor imagery with mental practice. This lack of clarity in terminology complicates the synthesis of evidence and may obscure the true effectiveness of these interventions. Indeed, our qualitative study (117) found that understanding of AO, MI, and AO + MI varied considerably among participants, reinforcing the need for consistent, clear terminology in research and clinical settings. Such variability in comprehension and language not only complicates assessment of efficacy but likely hinders intervention uptake, underscoring the importance of aligning clinical evidence with real-world user experience in future work.

Our review also revealed significant differences in the design, delivery and reporting of AO + MI interventions across studies. This reaffirms the ongoing issue regarding the lack of a standardized reporting of experimental protocols (107). The lack of standardization in how these interventions are applied, particularly concerning the motor simulation states they evoke, poses a major challenge to the comparability and generalizability of results. Future research should ensure consistent and precise terminology to improve comparability and clarify underlying mechanisms. Please see the check list for peer-reviewers as part of the Guidelines for Reporting Action Simulation Studies (107).

Diverse media and technologies, such as pictures, videos, virtual reality (VR), and BCI systems, have been employed in AO + MI interventions, further contributing to the proliferation in terminology and arrangements. This diversity in intervention approaches makes it difficult to draw definitive conclusions about the most effective methods for delivering AO + MI. To address this issue, future research should focus on developing standardized intervention protocols that can be reliably implemented across different settings and populations.

These factors, combined with the relatively small number of studies included in the present analysis, suggest that the results should be interpreted with some caution. Future research should aim to address these limitations by developing standardized and clearly defined intervention protocols and by increasing the consistency of terminology and outcome measures used in this field.

## 5 Conclusion

The findings of this study demonstrate that AO + MI can enhance both upper limb function and neuroimaging outcomes following stroke. The meta-analysis revealed a medium positive effect overall for AO + MI practice on FMA-UE and ARAT scores. Moreover, the narrative synthesis demonstrated how these effects are reported more widely outside of RCT designs and across neuroimaging outcomes. While AO + MI interventions show significant promise for improving upper limb function in stroke patients, this systematic review and meta-analysis emphasizes the importance of addressing key factors such as age, intervention duration, outcome measures, terminology, and standardization. Tackling these challenges in future research will be critical to fully realize the clinical potential of AO + MI and ensure its effective integration into clinical practice. In practice, we submit that AO + MI might be best suited to acting as a bridge between AO therapy, which requires little effort in early recovery, to the more effortful and advanced practice of independent MI, as a progressive training program toward improved motor execution.

## Data Availability

The original contributions presented in the study are included in the article/Supplementary material, further inquiries can be directed to the corresponding author/s.
