# Supplementary material for: Combined action observation and motor imagery practice for upper limb recovery following stroke: a systematic review and meta-analysis
Source: Front Neurol. 2025 Jul 23;16:1567421. doi: 10.3389/fneur.2025.1567421 (PMC12327397; doi:10.3389/fneur.2025.1567421)
Supplement: Supplementary file 1 [file Table_1.docx]

**CINAHL:**

https://www.ebsco.com/products/ebscohost-research-platform

Boolean operators used: OR, AND

Search Results: 37

(Stroke OR Cerebrovascular accident OR CVA OR Hemiplegia) AND (Motor imagery OR Guided imagery OR Imagery OR Visualisation OR Mental imagery OR Kinesthetics imagery OR Visual imagery OR Mental practice OR Mental training OR Mental rehearsal) AND (Mirror therapy OR Mirror OR Mirror neurone OR Action observation OR Action observation training OR Video therapy) AND (Upper extremity OR Upper limb OR Hand OR Hand function)

**MEDLINE:**

<https://www.ebsco.com/products/ebscohost-research-platform>

Boolean operators used: OR, AND

Search Results: 67

(Stroke OR Cerebrovascular accident OR CVA OR Hemiplegia) AND (Motor imagery OR Guided imagery OR Imagery OR Visualisation OR Mental imagery OR Kinesthetics imagery OR Visual imagery OR Mental practice OR Mental training OR Mental rehearsal) AND (Mirror therapy OR Mirror OR Mirror neurone OR Action observation OR Action observation training OR Video therapy) AND (Upper extremity OR Upper limb OR Hand OR Hand function)

**PsycINFO:**

https://www.ebsco.com/products/ebscohost-research-platform

Boolean operators used: OR, AND

Search Results: 30

(Stroke OR Cerebrovascular accident OR CVA OR Hemiplegia) AND (Motor imagery OR Guided imagery OR Imagery OR Visualisation OR Mental imagery OR Kinesthetics imagery OR Visual imagery OR Mental practice OR Mental training OR Mental rehearsal) AND (Mirror therapy OR Mirror OR Mirror neurone OR Action observation OR Action observation training OR Video therapy) AND (Upper extremity OR Upper limb OR Hand OR Hand function)

**AMED:**

https://www.ebsco.com/products/ebscohost-research-platform

Boolean operators used: OR, AND

Search Results: 6

(Stroke OR Cerebrovascular accident OR CVA OR Hemiplegia) AND (Motor imagery OR Guided imagery OR Imagery OR Visualisation OR Mental imagery OR Kinesthetics imagery OR Visual imagery OR Mental practice OR Mental training OR Mental rehearsal) AND (Mirror therapy OR Mirror OR Mirror neurone OR Action observation OR Action observation training OR Video therapy) AND (Upper extremity OR Upper limb OR Hand OR Hand function)

**Web of Science:**

https://www-webofscience-com./wos/woscc/advanced-search

Field tag used: All Fields

Boolean used: OR, AND

Search Results: 254

ALL=(Stroke OR Cerebrovascular accident OR CVA OR Hemiplegia) AND ALL=( Motor imagery OR Guided imagery OR Imagery OR Visualisation OR Mental imagery OR Kinesthetics imagery OR Visual imagery OR Mental practice OR Mental training OR Mental rehearsal) AND ALL=( Mirror therapy OR Mirror OR Mirror neurone OR Action observation OR Action observation training OR Video therapy) AND ALL=( Upper extremity OR Upper limb OR Hand OR Hand function)

**Embase:**

https://ovidsp.dc1.ovid.com/ovid-new-a/ovidweb.cgi

Field tag used: All Fields

Boolean used: OR, AND

Search Results: 99

((Stroke or Cerebrovascular accident or CVA or Hemiplegia) and (Motor imagery or Guided imagery or Imagery or Visualisation or Mental imagery or Kinesthetics imagery or Visual imagery or Mental practice or Mental training or Mental rehearsal) and (Mirror therapy or Mirror or Mirror neurone or Action observation or Action observation training or Video therapy) and (Upper extremity or Upper limb or Hand or Hand function)).af.

**PubMed:**

<https://pubmed.ncbi.nlm.nih.gov/advanced/>

Field tag used: All Fields

Boolean used: OR, AND

Search Results: 266

(((Stroke or Cerebrovascular accident or CVA or Hemiplegia) AND (Motor imagery or Guided imagery or Imagery or Visualisation or Mental imagery or Kinesthetics imagery or Visual imagery or Mental practice or Mental training or Mental rehearsal)) AND (Mirror therapy or Mirror or Mirror neurone or Action observation or Action observation training or Video therapy)) AND (Upper extremity or Upper limb or Hand or Hand function)

**Scopus:**

https://www-scopus-com./search/advanced

Field tag used: Article Title/Abstract/Keywords

Boolean used: OR, AND

Search Results: 102

( TITLE-ABS-KEY ( stroke OR "Cerebrovascular accident" OR cva OR hemiplegia ) AND TITLE-ABS-KEY ( "Motor imagery" OR "Guided imagery" OR imagery OR visualisation OR "Mental imagery" OR "Kinesthetics imagery" OR "Visual imagery" OR "Mental practice" OR "Mental training" OR "Mental rehearsal" ) AND TITLE-ABS-KEY ( "Mirror therapy" OR mirror OR "Mirror neurone" OR "Action observation" OR "Action observation training" OR "Video therapy" ) AND TITLE-ABS-KEY ( "Upper extremity" OR "Upper limb" OR hand OR "Hand function" ) )

**PEDro:**

https://search.pedro.org.au/advanced-search

Field tag used: Abstract and Title

Searching method: PEDro search interface

Total Search Results: 9

Keywords: Stroke "Mental practice" "Action observation"

Search Results: 3

Keywords: Stroke "Motor imagery" "Action observation"

Search Results: 6

**Cochrane library:**

https://www.cochranelibrary.com/advanced-search

Field tag used: Article Title/Abstract/Keywords

Boolean used: OR, AND

Search Results: 124

(Stroke or Cerebrovascular accident or CVA or Hemiplegia):ti,ab,kw AND (Motor imagery or Guided imagery or Imagery or Visualisation or Mental imagery or Kinesthetics imagery or Visual imagery or Mental practice or Mental training or Mental rehearsal):ti,ab,kw AND (Mirror therapy or Mirror or Mirror neurone or Action observation or Action observation training or Video therapy):ti,ab,kw AND (Upper extremity or Upper limb or Hand or Hand function):ti,ab,kw
