# Supplementary material for: Combined action observation and motor imagery practice for upper limb recovery following stroke: a systematic review and meta-analysis
Source: Front Neurol. 2025 Jul 23;16:1567421. doi: 10.3389/fneur.2025.1567421 (PMC12327397; doi:10.3389/fneur.2025.1567421)

**Results - Outlier Diagnostics and Influence Analysis**

Potential outliers and influential effect sizes were identified across the meta-analysis. The removal of these outliers and influential cases had a minimal impact on the pooled effect and heterogeneity estimates for the meta-analyses. Outlier diagnostics were completed using the FIND.OUTLIERS function to address concerns about between-study heterogeneity. One study (Page & Levine, 2021) was identified as an outlier in the meta-analysis. The removal of this outlier had minimal impact on the pooled effect and heterogeneity estimates (*d* = 0.58, Q = 7.70, *p* = 0.26).

Influence analyses were conducted using the ‘INFLUENCEANALYSIS’ function from the ‘dmetar’ package in R. This process involved visual inspection of the "Baujat" (see Figure 1) and "influence" plots for effect sizes (see Figure 2), as well as "leave-one-out" plots (see Figure 3) for both effect size and I² values. one effect size (Page & Levine, 2021) influential in the upper-limb function outcome data.

Therefore, the effect size of (Page & Levine, 2021) study was excluded from the meta-analysis as it was deemed an influential outlier, resulting in a substantial change to the pooled effect and heterogeneity when removed from the analysis. All other effect sizes were retained in the meta-analyses to maintain the integrity and richness of the data.

*Figure 1. Baujat Plot*


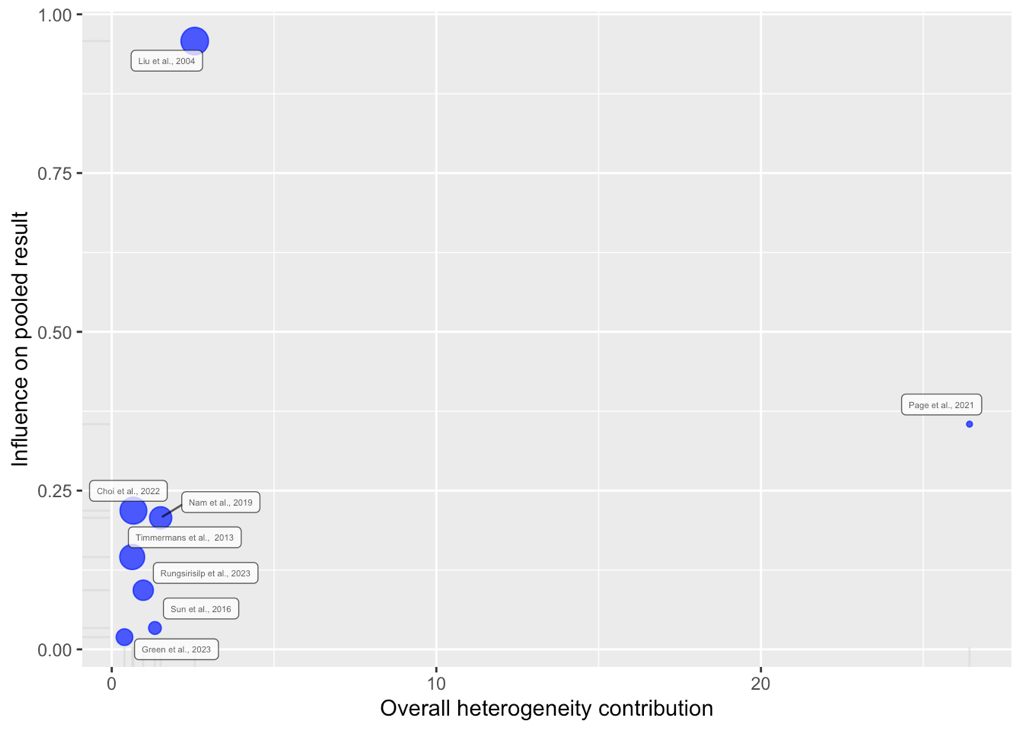


*Figure 2. “Influence” Plot*


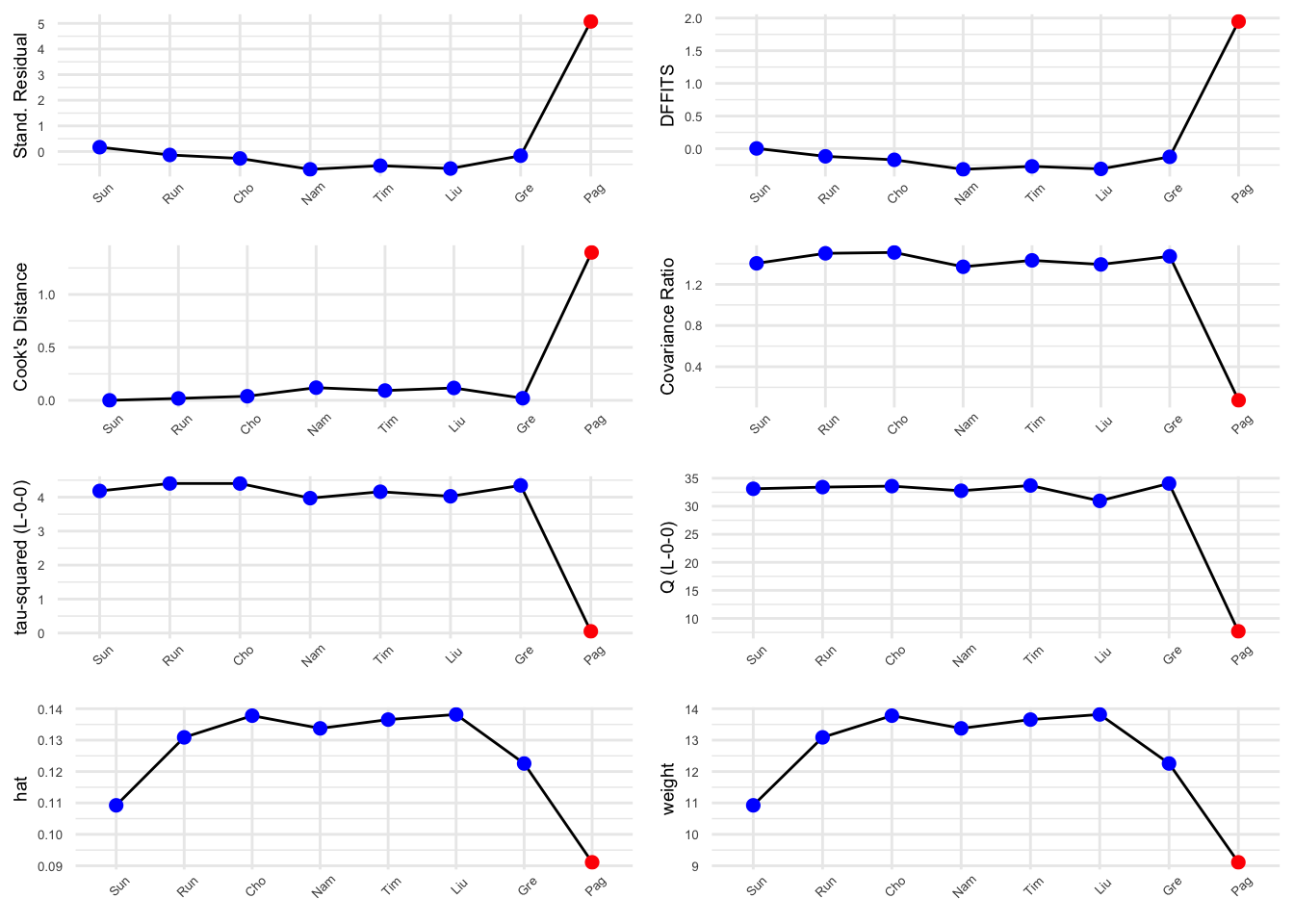


*Figure 2. "Leave-one-out" plots*


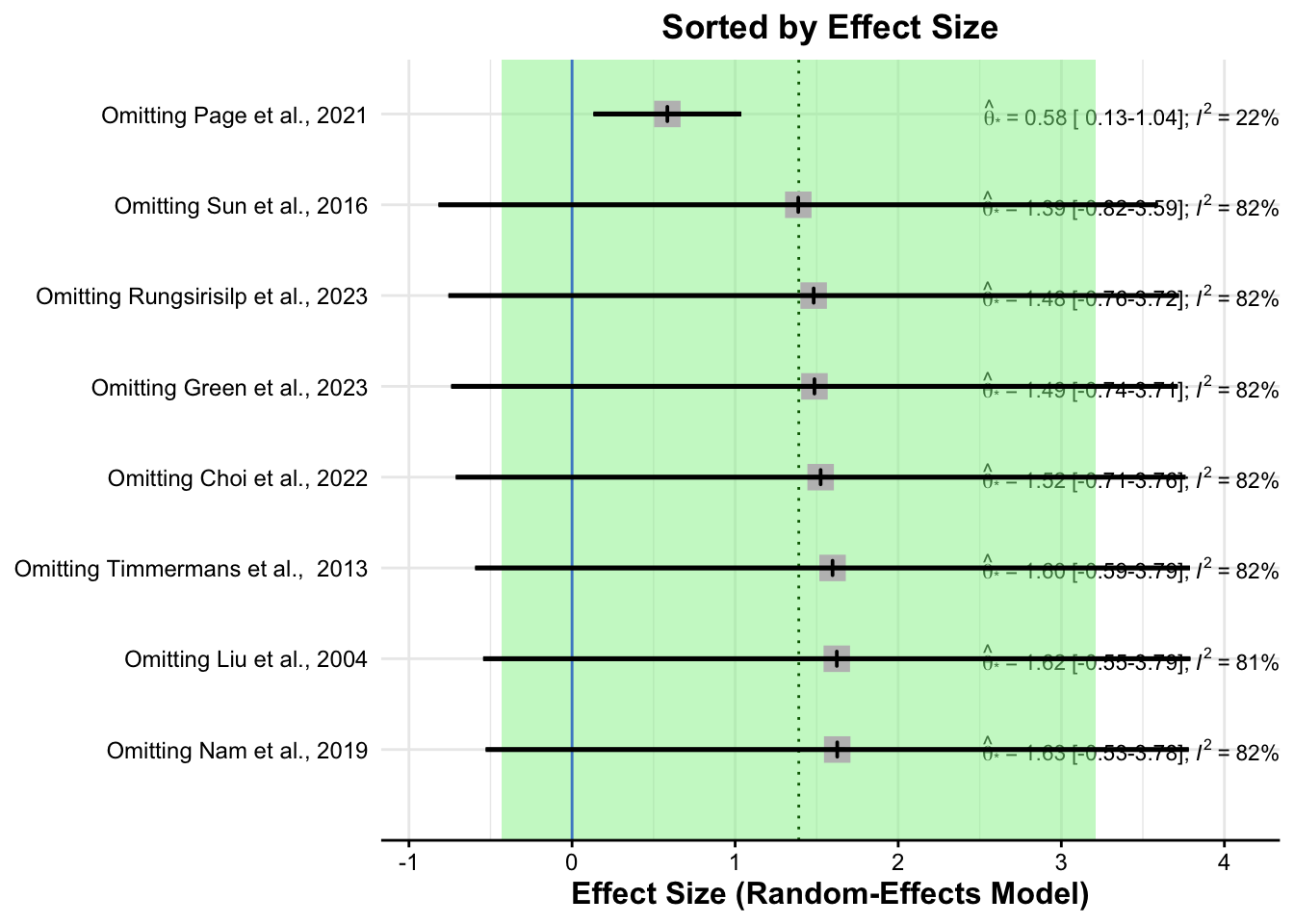

Supplement: Supplementary file 2 [file Table_2.docx]
