# Supplementary material for: Combined action observation and motor imagery practice for upper limb recovery following stroke: a systematic review and meta-analysis
Source: Front Neurol. 2025 Jul 23;16:1567421. doi: 10.3389/fneur.2025.1567421 (PMC12327397; doi:10.3389/fneur.2025.1567421)
Supplement: Supplementary file 3 [file Table_3.docx]

**Reference List for Included Studies**

***Reference List for Studies Included in the Meta-Analysis:***

Choi, J. B., Yang, S. W., & Ma, S. R. (2022). The Effect of Action Observation Combined with Motor Imagery Training on Upper Extremity Function and Corticospinal Excitability in Stroke Patients: A Randomized Controlled Trial. *International Journal of Environmental Research and Public Health*, *19*(19). <https://doi.org/10.3390/ijerph191912048>

Green, T. M., Fromm, N. M., Gayle, F. S., Lee, J., Wang, W., & Vas, A. K. (2023). Examining the Delivery Mode of Mental Practice in Reducing Hemiparesis: A Randomized Controlled Trial. *The Open Journal of Occupational Therapy*, *11*(4), 1–9. <https://doi.org/10.15453/2168-6408.2149>

Liu, H., Song, L. P., & Zhang, T. (2014). Mental practice combined with physical practice to enhance hand recovery in stroke patients. *Behavioural Neurology*, *2014*. <https://doi.org/10.1155/2014/876416>

Liu, K. P., Chan, C. C., Lee, T. M., & Hui-Chan, C. W. (2004). Mental imagery for promoting relearning for people after stroke: A randomized controlled trial. *Archives of Physical Medicine and Rehabilitation*, *85*(9), 1403–1408. <https://doi.org/10.1016/j.apmr.2003.12.035>

Nam, J. S., Yi, T. I., & Moon, H. I. (2019). Effects of adjuvant mental practice using inverse video of the unaffected upper limb in subacute stroke: a pilot randomized controlled study. *International Journal of Rehabilitation Research*, *42*(4), 337–343. <https://doi.org/10.1097/MRR.0000000000000368>

Rungsirisilp, N., Chaiyawat, P., Techataweesub, S., Meesrisuk, A., & Wongsawat, Y. (2023). Applying Action Observation During a Brain-Computer Interface on Upper Limb Recovery in Chronic Stroke Patients. *IEEE Access*, *11*(December 2022), 4931–4943. <https://doi.org/10.1109/ACCESS.2023.3236182>

Sun, Y., Wei, W., Luo, Z., Gan, H., & Hu, X. (2016). Improving motor imagery practice with synchronous action observation in stroke patients. *Topics in Stroke Rehabilitation*, *23*(4), 245–253. <https://doi.org/10.1080/10749357.2016.1141472>

Thara, N. (2015). Comparative Study Between Task Specific Motor Imagery With Mental Practice Versus Task Specific Mirror Therapy on Upper Limb Functions for Sub Acute Hemiplegia. *International Journal of Physiotherapy*, *2*(5), 824–833. <https://doi.org/10.15621/ijphy/2015/v2i5/78241>

Timmermans, A. A. A., Verbunt, J. A., van Woerden, R., Moennekens, M., Pernot, D. H., & Seelen, H. A. M. (2013). Effect of Mental Practice on the Improvement of Function and Daily Activity Performance of the Upper Extremity in Patients With Subacute Stroke: A Randomized Clinical Trial. *Journal of the American Medical Directors Association*, *14*(3), 204–212. <https://doi.org/10.1016/j.jamda.2012.10.010>

***Reference List for Studies Included in the Narrative Review:***

Binks, J. A., Emerson, J. R., Scott, M. W., Wilson, C., van Schaik, P., & Eaves, D. L. (2023). Enhancing upper-limb neurorehabilitation in chronic stroke survivors using combined action observation and motor imagery therapy. *Frontiers in Neurology*, *14*. <https://doi.org/10.3389/fneur.2023.1097422>

Cha, Y. J., Yoo, E. Y., Jung, M. Y., Park, S. H., Park, J. H., & Lee, J. (2015). Effects of mental practice with action observation training on occupational performance after stroke. *Journal of Stroke and Cerebrovascular Diseases*, *24*(6), 1405–1413. <https://doi.org/10.1016/j.jstrokecerebrovasdis.2015.02.022>

Fujiwara, K., Shimoda, R., Shibata, M., Awano, Y., Shibayama, K., & Higashi, T. (2023). A Method for Using Video Presentation to Increase Cortical Region Activity during Motor Imagery Tasks in Stroke Patients. *Brain Sciences*, *13*(1). <https://doi.org/10.3390/brainsci13010029>

Huang, J., Lin, M., Fu, J., Sun, Y., & Fang, Q. (2021). An Immersive Motor Imagery Training System for Post-Stroke Rehabilitation Combining VR and EMG-based Real-Time Feedback. *Proceedings of the Annual International Conference of the IEEE Engineering in Medicine and Biology Society, EMBS*, 7590–7593. <https://doi.org/10.1109/EMBC46164.2021.9629767>

Ichidi, A., Hanafusa, Y., Itakura, T., & Tanaka, T. (2018). Simultaneous Observation and Imagery of Hand Movement Enhance Event-Related Desynchronization of Stroke Patients. In J. M. Delgado-García, X. Pan, R. Sánchez-Campusano, & R. Wang (Eds.), *Advances in Cognitive Neurodynamics (VI)* (pp. 71–77). Springer Singapore.

Lin, M., Huang, J., Fu, J., Sun, Y., & Fang, Q. (2023). A VR-Based Motor Imagery Training System With EMG-Based Real-Time Feedback for Post-Stroke Rehabilitation. *IEEE Transactions on Neural Systems and Rehabilitation Engineering*, *31*, 1–10. <https://doi.org/10.1109/TNSRE.2022.3210258>

Robinson-Bert, K., & Woods, A. B. (2022). Effectiveness of synchronous action observation and mental practice on upper extremity motor recovery after stroke. *Occupational Therapy in Health Care*, *38*(2), 196–213. <https://doi.org/10.1080/07380577.2022.2138675>

Rungsirisilp, N., & Wongsawat, Y. (2022). Applying Combined Action Observation and Motor Imagery to Enhance Classification Performance in a Brain-Computer Interface System for Stroke Patients. *IEEE Access*, *10*(June), 73145–73155. <https://doi.org/10.1109/ACCESS.2022.3190798>

Sun, Y., Yin, L., Meng, M., Ma, Y., Luo, Z., & Bao, F. S. (2014). Facilitating motor imagery practice with synchronous action observation for stroke patients. *2014 IEEE Healthcare Innovation Conference, HIC 2014*, 231–235. <https://doi.org/10.1109/HIC.2014.7038917>
